# Inhibition of toxic metal-alpha synuclein interactions by human serum albumin†

**DOI:** 10.1039/d3sc06285f

**Published:** 2024-01-31

**Authors:** Karla Martinez Pomier, Rashik Ahmed, Jinfeng Huang, Giuseppe Melacini

**Affiliations:** a Department of Chemistry and Chemical Biology, McMaster University ON L8S 4M1 Canada melacin@mcmaster.ca; b Department of Biochemistry and Biomedical Sciences, McMaster University Hamilton ON L8S 4M1 Canada

## Abstract

Human serum albumin (HSA), the most abundant protein in plasma and cerebrospinal fluid, not only serves as a crucial carrier of various exogenous and endogenous ligands but also modulates the aggregation of amyloidogenic proteins, including alpha synuclein (αSyn), which is associated with Parkinson's disease and other α-synucleinopathies. HSA decreases αSyn toxicity through the direct binding to monomeric and oligomeric αSyn species. However, it is possible that HSA also sequesters metal ions that otherwise promote aggregation. Cu(ii) ions, for example, enhance αSyn fibrillization *in vitro*, while also leading to neurotoxicity by generating reactive oxygen species (ROS). However, it is currently unclear if and how HSA affects Cu(ii)-binding to αSyn. Using an integrated set of NMR experiments, we show that HSA is able to chelate Cu(ii) ions from αSyn more efficiently than standard chelators such as EDTA, revealing an unexpected cooperativity between the HSA metal-binding sites. Notably, fatty acid binding to HSA perturbs this cooperativity, thus interfering with the sequestration of Cu(ii) ions from αSyn. We also observed that glycation of HSA diminished Cu(ii)-binding affinity, while largely preserving the degree of cooperativity between the HSA metal-binding sites. Additionally, our results show that Cu(ii)-binding to HSA stabilizes the interactions of HSA with αSyn primarily at two different regions, *i.e.* the N-terminus, Tyr 39 and the majority of the C-terminus. Our study not only unveils the effect of fatty acid binding and age-related posttranslational modifications, such as glycation, on the neuroprotective mechanisms of HSA, but also highlights the potential of αSyn as a viable NMR-based sensor to investigate HSA-metal interactions.

## Abstract 2

Human serum albumin (HSA) not only serves as a crucial carrier of various ligands but also modulates the aggregation of amyloidogenic proteins, including alpha synuclein (αSyn), which is associated with Parkinson's disease and other synucleinopathies.

## Graphical abstract

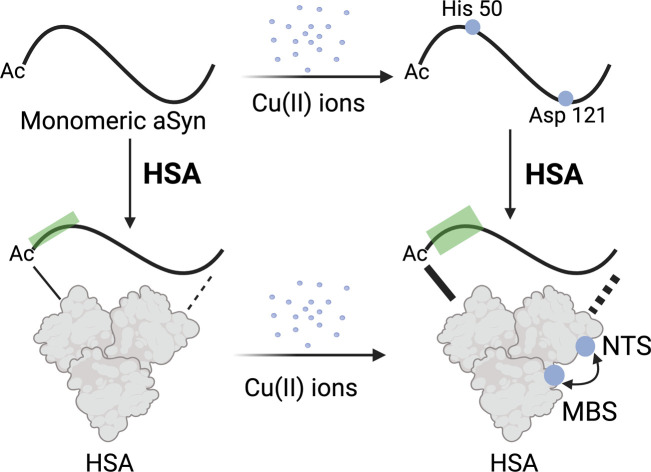

## Introduction

**Fig. 1:**
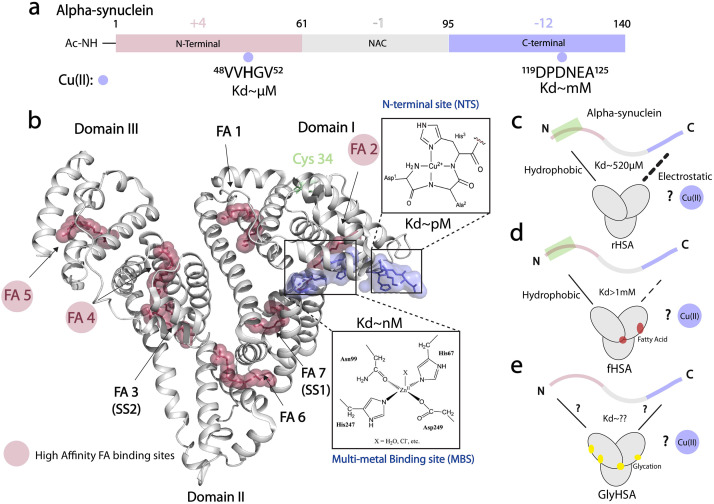
Cu(ii) binding properties of HSA, and monomeric alpha synuclein. (a) Monomeric alpha synuclein is comprised of three distinct regions: a positively charged N-terminal region spanning residues 1–60 (red), the non-amyloid-β component (NAC) region spanning residues 61–95 (grey), and the negatively charged C-terminal region spanning residues 96–140 (light purple). Cu(ii), represented by light purple spheres, binds N-terminal acetylated αSyn (Ac-αSyn) at two different anchoring sites: the low affinity, non-specific metal binding site at the acidic DPDNEA segment, and the high affinity site at His-50. (b) Crystal structure of oleic acid bound HSA (PDB code: 1GNI). Oleic acid is shown as red spheres. High affinity fatty-acid binding sites are highlighted with a red circle (FA 2, FA 4 and FA 5), whereas low affinity sites are labelled in black. Cu(ii) binding to HSA involves the ATCUN (amino terminal copper and nickel) binding site or N-terminal binding site (NTS). Cu(ii) can also bind to the multi-metal binding site (MBS), but under physiological conditions, the MBS site is mostly occupied by Zn(ii). This structure was generated by merging the HSA crystal structure (PDB code: 1GNI) and the crystal structure of NTS model peptide DAKH complexed with Cu(ii) ions (CCDC-809109) (c) HSA binds αSyn monomers at the N- and C-termini through hydrophobic and electrostatic interactions, respectively. (d) The binding at the C-terminus is compromised upon FA binding to HSA. (e) The role that glycation of HSA plays in HSA-αSyn interactions is unknown. (c–e) The role of Cu(ii) ions in the αSyn-HSA complex is also unknown.

Protein aggregates known as Lewy bodies (LB) are the hallmark of Parkinson's disease (PD).^1,2^ One of the main components of LB is alpha-synuclein (αSyn), an intrinsically disordered protein (IDP) that is predominantly expressed in the brain and self-associates into toxic aggregates and amyloid fibrils.^3–12^ The primary structure of αSyn spans 140 amino acids and is divided into three main regions: the N-terminal region (NTR, residues 1–60), which is positively charged and is important for lipid, chaperone, and lipopolysaccharide^11^ binding; the mainly hydrophobic non-amyloid-β component region (NAC, residues 61–95), which is critical for fibril development; and, the C-terminal region (CTR, residues 96–140), which is negatively charged and can bind various ligands including proteins, small molecules, and metal ions (Fig. 1a).^13–24^ Among the latter, copper ions have garnered attention for their potential to generate reactive oxygen species (ROS).^14,19–22^ Physiologically-relevant N-terminally acetylated αSyn (Ac-αSyn) interacts with Cu(ii) ions with medium affinity (∼μM) at the His 50 site^14,25^ and binds weakly (mM) and non-specifically most metal ions at the acidic DPDNEA segment in the CTR^14,24,26^ (Fig. 1a). The His 50 site has been linked to αSyn fibrillization through the familial PD mutation H50Q and is also a potential target for physiological amyloid inhibitors such as the small molecule heme.^27^

Although considered predominantly intracellular, αSyn is also located extracellularly in cerebrospinal fluid (CSF) and blood plasma.^28^ Extracellular αSyn leads to cell-to-cell transmission of synucleinopathies *via* a prion-like mechanism.^29,30^ Additionally, once in the extracellular space, αSyn is exposed to CSF and plasma components that can perturb its structure and its later entry to cells, triggering not only oligomer and amyloid formation but also the progressive spreading of Lewy body diseases and neuroinflammation.^29^ Furthermore, extracellular αSyn binds endogenous chaperones, such as human serum albumin (HSA),^31,32^ the most abundant protein in blood plasma (∼640 μM) and CSF (∼3 μM) and a potent amyloid inhibitor of several IDPs, including αSyn^10,33–36^ and the Aβ peptide associated with Alzheimer's disease (AD).^32,37–45^

HSA inhibits αSyn toxicity through several mechanisms. One of the mechanisms involves the direct binding of HSA to oligomeric αSyn species through hydrophobic interactions, which remodel oligomers into off-pathway chimeric assemblies with decreased cellular toxicity.^46^ HSA can also disrupt the interaction of toxic αSyn oligomers with membranes, thus inhibiting the insertion of αSyn oligomeric species into lipid bilayers and the consequent loss of membrane integrity. Furthermore, at plasma concentrations, HSA inhibits αSyn's early aggregation by binding αSyn monomer's N- and C-terminal sites (Fig. 1c, top), although the latter interaction is weakened by long-chain fatty acid binding to HSA.^46^

Besides these amyloid inhibition mechanisms, HSA is an endogenous chelator of several metal ions, that are related to neurogenerative disorders, including PD and AD.^47–50^ Cu(ii) and Zn(ii) ions are essential in brain neurobiology and their homeostasis has been found to be altered in several neurodegenerative diseases.^14,51–57^ HSA prevents Cu(ii)-induced Aβ aggregation by rapidly removing the ions stoichiometrically from the peptide.^58^ Previous reports also indicate that after chelating Zn(ii) and Cu(ii) ions,^42^ HSA conserves its binding to Aβ monomers and oligomers.

At physiological concentrations, Cu(ii) enhances αSyn fibrillization^14^ and Cu(ii) dys-homeostasis leads to neurotoxicity not only by promoting αSyn aggregation but also by generating ROS.^22^ HSA can bind up to four equivalents of Cu(ii);^59^ two sites bind the ion specifically *i.e.* the N-terminal site (NTS, *K*_d_ ∼ pM; Fig. 1b) and the multi-metal binding site (MBS, *K*_d_ ∼10 nM; Fig. 1b). A third site, site B, with an unknown location is predicted to also bind Cu(ii) ions but with reduced affinity (∼μM) compared to the NTS and MBS.^60^

While sequestration of Cu(ii) ions from αSyn by HSA provides an effective neuroprotective mechanism, it is not yet clear whether and how HSA can bind Cu(ii) ions originally bound to αSyn. It is also unknown whether the binding of monomeric αSyn with HSA is affected in the presence of metal ions (Fig. 1c). Additionally, previous reports of Cu(ii) transfer from amyloidogenic proteins to HSA, did not take into account posttranslational modifications of HSA such as glycation, or the effect of physiological ligands such as long chain fatty acids (Fig. 1d and e). Here, we fill these gaps by using an integrated set of NMR experiments.

We show that HSA is able to sequester Cu(ii) ions from Ac-αSyn more efficiently than standard chelators such as EDTA, revealing what is to our knowledge unprecedented evidence of cooperativity between HSA's Cu(ii) binding sites. Our data suggests that fatty acid binding to HSA perturbs the cooperativity between its metal binding sites, therefore interfering with the chelation of Cu(ii) ions from αSyn. Glycated HSA showed diminished binding affinity to Cu(ii) compared to non-modified HSA, but the cooperativity between the Cu(ii)-binding sites was largely conserved. Additionally, we found that while fatty acid-bound HSA interacts with acetylated αSyn similarly to non-acetylated αSyn,^46^ glycated HSA abolishes binding at αSyn's C-terminus but not at the N-terminus. Furthermore, Cu(ii)-bound HSA enhances binding to αSyn at both the N- and C-terminal regions. Our findings also highlight the potential of αSyn-NMR as a viable sensor to investigate the interactions of metal ions with HSA.

## Results and discussion

### HSA sequesters Cu(ii) ions from both Ac-αSyn metal binding sites and is a more potent chelator than EDTA

**Fig. 2:**
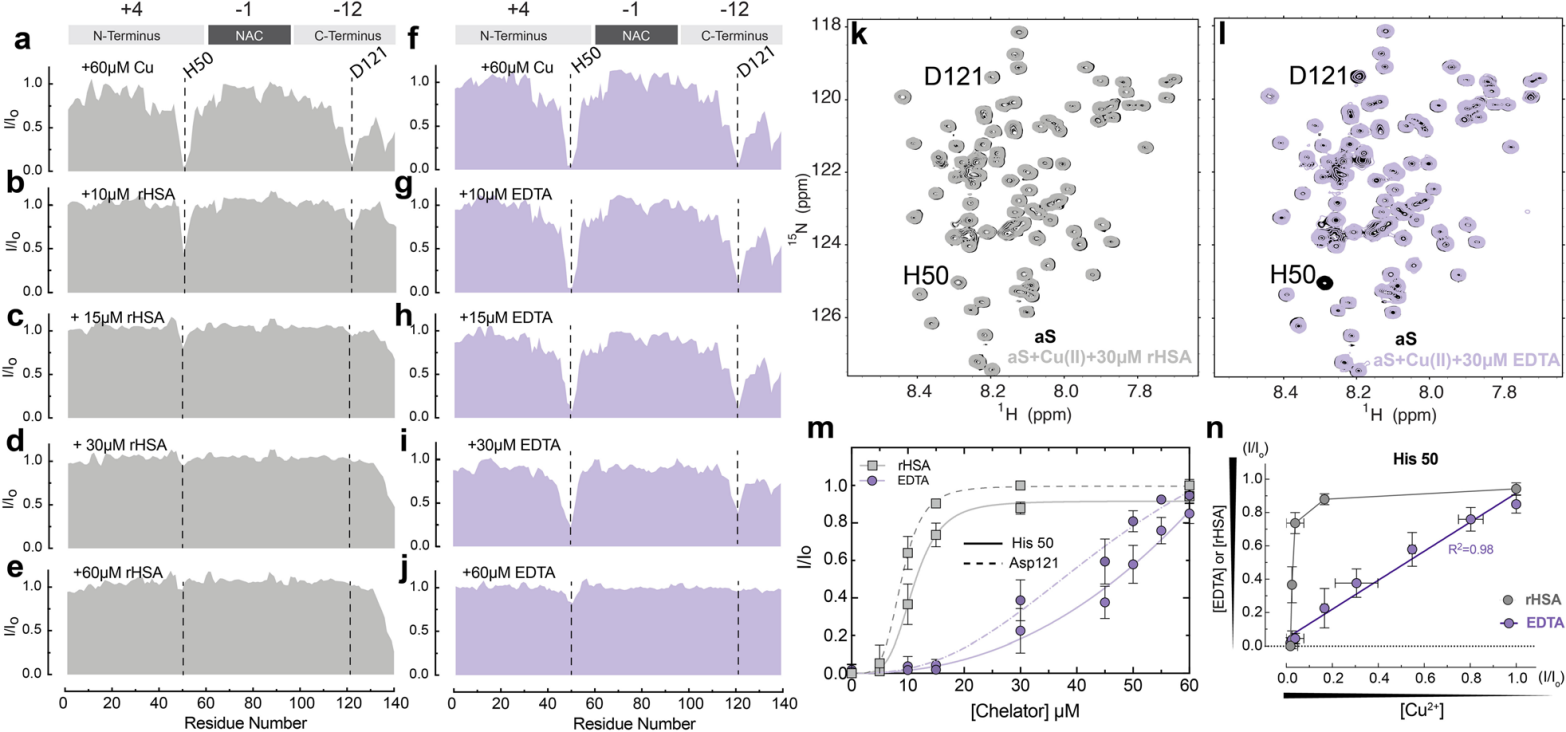
HSA chelates Cu(ii) more efficiently than the standard chelator EDTA. (a–e) Normalized sfHMQC cross-peak intensities (*I*/*I*_o_) as a function of residue number for 60 μM Ac-αSyn in the presence of 60 μM Cu(ii) (a) and increasing concentrations of rHSA (b–e). (f–j) Same as (a–e) but with increasing concentrations of EDTA. (k) Overlaid contour plots of the ^1^H–^15^N sfHMQC spectra of 60 μM aSyn (black) and 60 μM aSyn in the presence of 60 μM Cu(ii) and 30 μM rHSA (grey). (l) As (k) with rHSA replaced by 30 μM EDTA (lilac). (m) Isolated I/*I*_o_ profiles for residues His-50 (solid lines) and Asp-121 (dashed lines) plotted against increasing concentrations of rHSA (grey) and EDTA (lilac) respectively. Experimental points were fitted to a Hill-like model. (n) *I*/*I*_o_ profiles for the αSyn-Cu(ii) binding site at His-50 upon chelation of Cu(ii) ions with increasing concentrations of EDTA (lilac) or rHSA (grey) *vs.* the *I*/*I*_o_ observed upon addition of the corresponding amount of remaining αSyn-bound Cu(ii), assuming a binding stoichiometry of 1 : 1 for Cu(ii) : EDTA and 2 : 1 for Cu(ii) and rHSA. Spectra were acquired at 10 °C in 50 mM HEPES, pH 7.4. Error bars represent the standard deviation of three neighbouring residues.

To probe at residue resolution metal – Ac-αSyn interactions and metal chelation by HSA we relied on Band-Selective Optimized Flip Angle Short Transient (SO-FAST) Heteronuclear Multiple Quantum Coherence (HMQC) experiments, which allow the recording of 2D NMR spectra for ^15^N-labeled Ac-αSyn with high sensitivity and resolution.^61^ The normalized sfHMQC intensity profiles of 60 μM Ac-αSyn in the presence of equimolar concentrations of Cu(ii) indicate two major regions of signal losses centered at His-50 and Asp-121 (Fig. 2a and S1a†), as expected based on the binding of paramagnetic Cu(ii) ions at both sites (Fig. 1a).^14,62,63^ When unlabeled fatty-acid free HSA (rHSA) was titrated into the equimolar solution of Ac-αSyn and Cu(ii) ions, we observed a progressive recovery of intensities at both His-50 and Asp-121 sites (Fig. 2b–e), indicating that rHSA removes Cu(ii) from Ac-αSyn. Notably, rHSA sequesters Cu(ii) more readily away from the C-terminal binding site Asp-121 compared to the His-50 site (Fig. 2b, c, and m). This result is in agreement with previous reports for non-acetylated αSyn^62^ and can be explained by the higher affinity of the His-50 site for Cu(ii) ions (∼μM) compared to the C-terminal site (∼mM). In addition, we detected essentially a full Ac-αSyn signal recovery when rHSA reached half the concentration of Ac-αSyn and Cu(ii) ions (60 μM; Fig. 2d and k), indicating that a complete sequestration of Cu(ii) ions away from Ac-αSyn does not require equimolar rHSA concentrations. This result can be explained by rHSA's ability to coordinate two Cu(ii) ions at two different binding sites (*e.g.* NTS and MBS; Fig. 1b), although we cannot rule out potential contributions from site B at this stage. In contrast, the sequestration of Cu(ii) ions using the chemical chelator EDTA, which can only coordinate a single Cu(ii) ion, required equimolar EDTA concentrations (Fig. 2f–j, l, and m).

To assess whether the higher affinity of Cu(ii) for HSA (nM–pM) *vs.* Ac-αSyn (mM–μM) is the driving force of HSA chelation we conducted a control experiment with the paramagnetic ion Mn(ii). HSA binds Mn(ii) with lower affinity than Cu(ii), in the sub mM range,^64–66^ so it is possible that HSA might not chelate paramagnetic Mn(ii) ions from Ac-αSyn with the same efficacy. As expected, at equimolar concentrations of Ac-αSyn : Mn(ii) : rHSA, albumin was not able to completely recover the loss of αS intensities caused by Mn(ii) (S1c, d & S2†), hinting to its inability to fully sequester Mn(ii) ions away from Ac-αSyn.

Interestingly, besides the different Cu(ii) sequestration stoichiometries, the comparison of the sfHMQC intensity profiles acquired for the rHSA and EDTA titrations (Fig. 2m) reveals another major difference between these two chelators. rHSA exhibits a clearly sigmoidal pattern which is largely lost for EDTA (Fig. 2m). Such differential cannot be explained simply by the different Cu(ii)-binding stoichiometries of rHSA and EDTA, as scaling the rHSA concentrations does not recapitulate the EDTA intensity profiles (Fig. S3a–d†). Even after adjusting the concentration of HSA by a factor of two or three to take into consideration the distinct HSA and EDTA Cu(ii)-binding stoichiometries, albumin is still more efficient in chelating Cu(ii) ions than EDTA. This is observed for both Ac-αSyn binding sites, His-50 and Asp-121 (Fig. S3a–d†). Furthermore, the sigmoidal intensity pattern observed for rHSA (Fig. 2m) cannot be rationalized by the non-linear dependence of sfHMQC intensity losses upon binding of Cu(ii) to Ac-αSyn (Fig. S3e†). Such non-linearity is sufficient to account only for the non-linear intensity profile observed in the titration of EDTA (Fig. 2m). If the *I*/*I*_o_ ratios observed upon chelation of Cu(ii) ions by EDTA are plotted *vs.* the corresponding *I*/*I*_o_ ratios measured upon addition of the remaining Cu(ii) ions bound to Ac-αSyn after EDTA addition, a linear relationship is observed for both Ac-αSyn Cu(ii) binding sites (Fig. 2n and S3f†). Such a linear relationship indicates that the EDTA added at each step of the titration is quantitatively saturated with bound Cu(ii) sequestered away from Ac-αSyn. However, for rHSA, we did not observe a linear correlation (Fig. 2n and S3f†), confirming that the sigmoidal Ac-αSyn intensity profile observed for the rHSA titration (Fig. 2m) does not simply reflect the non-linear dependence of sfHMQC intensities on the concentration of residual non-sequestered Cu(ii). Overall, our data rule out Cu(ii)-binding stoichiometries or non-linear sfHMQC intensity dependencies on [Cu(ii)] as possible explanations of the sigmoidal shape observed for the intensity recovery of Cu(ii)-bound Ac-αSyn upon rHSA titration (Fig. 2m), suggesting that such sigmoidal pattern genuinely reflects positive cooperativity between the Cu(ii)-binding sites of rHSA. To our knowledge, this represents an unanticipated account of cooperativity between the Cu(ii) binding sites of HSA and highlights that Ac-αSyn can serve as an excellent indirect reporter of Cu(ii)–rHSA interactions.

Our results are also relevant in the context of PD and Cu(ii) neurotoxicity, as they show that due to HSA's high affinity for Cu(ii) ions and the positive cooperativity, HSA can effectively sequester Cu(ii) ions, thus avoiding their aberrant interactions with αSyn. Cu(ii) binding to extracellular Ac-αSyn and its sequestration by HSA is relevant for PD. Cu(ii) concentrations are elevated in the brain of PD patients or during localized events, such as the synaptic release of large amounts of Cu(ii) ions (∼20 μM), which can exceed CSF concentrations of HSA (∼3 μM).^67,68^ Additionally, Cu(ii)-αSyn binding can be relevant for PD patients for whom serum albumin levels are strikingly decreased compared to healthy individuals.^69,70^ On the same note, albumin levels decrease during aging, which represents a risk factor for neurodegenerative diseases like PD and AD.^71^

### The binding of long-chain fatty acids to HSA perturbs Cu(ii) sequestration from Ac-αSyn by reducing the cooperativity between albumin's metal binding sites

**Fig. 3:**
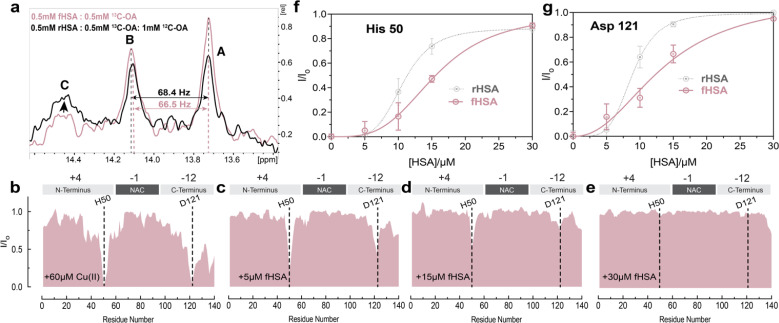
Fatty acid bound HSA displays decreased chelating ability compared to defatted HSA. (a) 1D-^13^C spectra of ^13^C-methyl labelled oleic acid in the presence of FA-free HSA (black) and fatty acid bound-HSA (pink). Peaks A and B represent the two highest affinity binding sites in HSA, while peak C represents the third highest affinity site, as previously reported (72). (b–e) Normalized sfHMQC cross-peak intensities (*I*/*I*_o_) as a function of residue number for 60 μM Ac-αSyn in the presence of 60 μM Cu(ii) and increasing concentrations of fHSA (red). Spectra were acquired at 10 °C in 50 mM HEPES, pH 7.4. (f and g) Isolated *I*/*I*_o_ profiles for residues His-50 and Asp-121 plotted against increasing concentrations of fHSA (red), and rHSA as a reference (grey dashed lines). Experimental points were fitted to a Hill-like model. Error bars represent the standard deviation of three neighbouring residues.

The binding of long-chain fatty acids (LCFAs) modulates HSA interactions with other ligands, including metal ions.^72^ Albumin contains seven binding sites for LCFAs scattered among the three domains with FA 2, 4, and 5 showing the highest affinities (Fig. 1b). Hence, we tested whether the binding of LCFAs would affect HSA's ability to chelate Cu(ii) ions. The saturation of HSA with LCFAs such as oleic acid leads to a rotation of domains one and three relative to domain two. We can use this inter-domain rearrangement to characterize and assess the number of endogenous LCFAs bound to non-defatted HSA (fHSA) using ^13^C-NMR. For this analysis, we relied on a previously described method called “^13^C-oleic acid for the NMR-based assessment of albumin-bound LCFA concentration” (CONFA).^73^ CONFA is based on the 1D-NMR properties of exogenous ^13^C-oleic acid bound to albumin.^74^ When HSA is already pre-bound to endogenous LCFAs, what we denote as fHSA, the addition of ^13^C-oleic acid drives an allosteric interdomain arrangement which results in NMR chemical shifts and intensity changes of albumin-bound ^13^C-oleic acid. Specifically, pre-bound LCFAs to HSA alter the intensity and/or frequencies of peaks A-C in the 1D-^13^C spectra of ^13^C-oleic acid (Fig. 3a). The frequency separation between peaks A and B (Δ*v*_AB_) can be used to estimate the amount of pre-bound LCFAs to HSA given that Δ*v*_AB_ is linearly correlated with the [^12^C-FA]_Tot_/[HSA]_Tot_ ratio (*r*)

1 Δ*v*_AB_ = *α* − *βr*

where *α* = 71.287 and *β* = 1.869 (*R*^2^ = 0.97) as previously estimated.^73^ Through eqn (1) we can estimate the stoichiometric ratio (*r*) for the total LCFAs initially bound to HSA preceding the addition of ^13^C oleic acid.

2 *r* = (*α* − Δ*v*_AB_)/*β*

Characterization of fHSA through CONFA revealed that it is bound to ∼one-two LCFA molecules per HSA molecule, based on a peak A–B separation of 68.4 Hz upon the addition of ^13^C-oleic acid (Fig. 3a).

To investigate if the binding of LCFAs to HSA affects its capacity to sequester Cu(ii) ions from Ac-αSyn, we repeated the NMR-monitored titration shown in Fig. 2a–e but with fatty-acid bound HSA (fHSA). A close inspection of both Ac-αSyn metal binding sites, His-50 and Asp-121 revealed a decreased chelating potency of fHSA in comparison to rHSA (Fig. 3f and g). Furthermore, the sigmoidal character of the *I*/*I*_o_ recovery observed for rHSA is now largely lost, suggesting that the cooperativity between the Cu(ii) metal binding sites in HSA decreases upon binding of LCFAs to albumin (Fig. 3f, g and S4†). In contrast to rHSA, Fig. S4c and d† shows that for fHSA scaling the concentration largely recapitulates the chelation profile observed for EDTA. These results are in agreement with previous reports of LCFAs modulating the interaction of HSA with Co(ii) and Zn(ii) ions, which share the MBS with Cu(ii). Binding of fatty acids allosterically inhibits the binding of Co(ii) and Zn(ii) ions to the MBS and the not-yet localized site B.^75–77^ Our data suggest that the reduced cooperativity between the HSA metal binding sites may also contribute to the shedding of metal ions upon binding of LCFAs, a phenomenon relevant to the detection of myocardial ischemia.^77^

### HSA glycation impairs Cu(ii) sequestration from Ac-αSyn by reducing the affinity of albumin's metal binding sites, while largely preserving positive cooperativity

Non-enzymatic glycation is a spontaneous post-translational modification of albumin. Due to its high concentration in plasma and CSF, HSA accounts for ∼80% of all glycated proteins in the body.^78^ While HSA is glycated in 1–10% of healthy individuals, during aging or diabetes where the level of blood glucose and other reducing sugars increases, this percentage can increase two-three fold.^78–81^ HSA modifications by physiologically relevant glycating agents such as glucose and methyl glyoxal lead to changes in the structure and ligand binding capabilities of the chaperone.^79,82–85^ Hence, we hypothesized that albumin glycation may also affect the sequestration of Cu(ii) ions away from Ac-αSyn and/or the cooperativity displayed by the metal binding sites of albumin. To test our hypothesis, we used an *in vitro* model of methyl-glyoxal (MGO) glycated HSA (GlyHSA).^86^

**Fig. 4:**
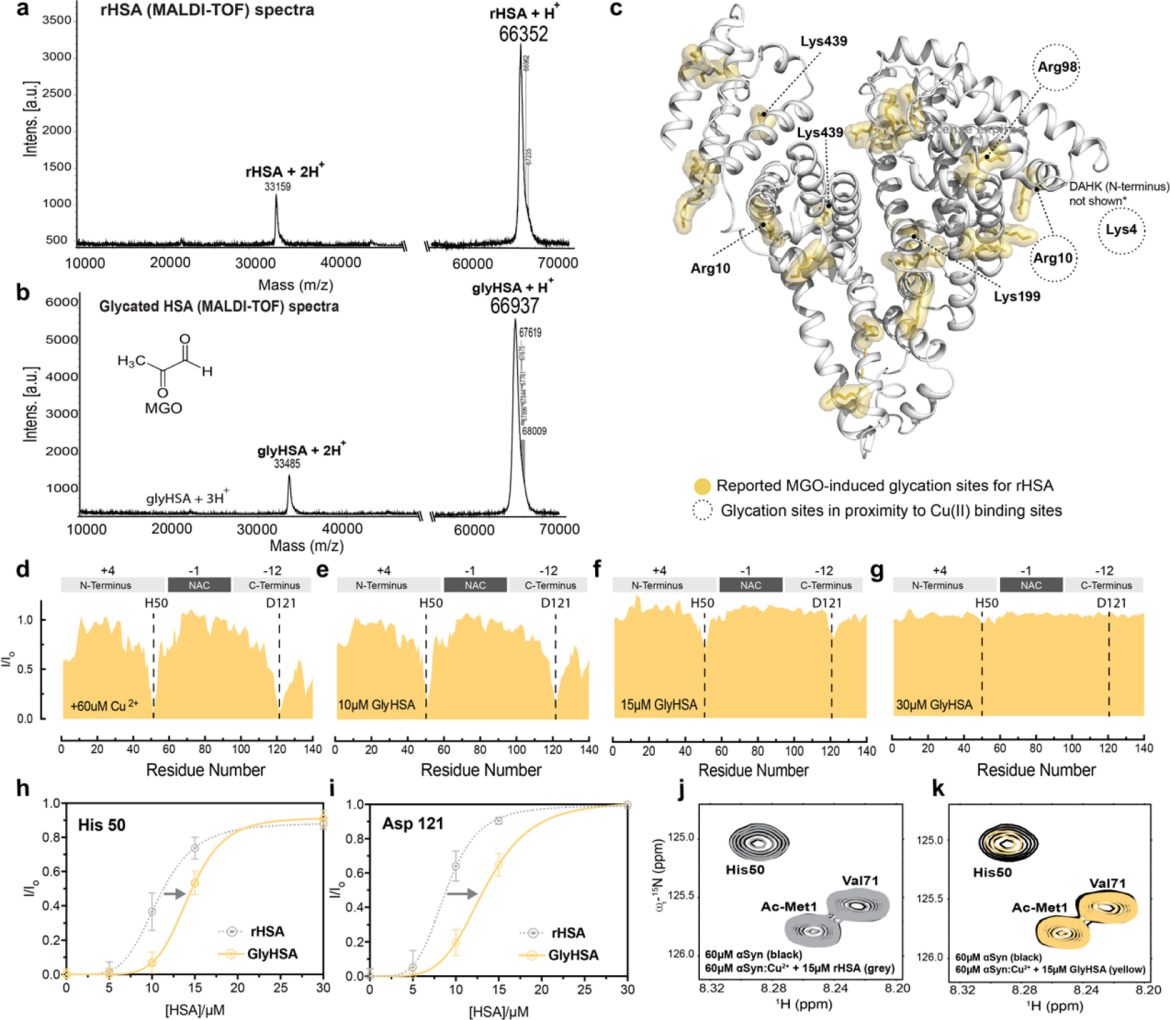
MGO-glycated HSA displays decreased Cu(ii) chelating ability compared to unmodified HSA. (a) MALDI-TOF MS of rHSA. (b) MALDI-TOF spectra of MGO-glycated HSA (GlyHSA). (c) 27 glycation sites on MGO-glycated HSA based on a previous report.^85^ (d–g) Normalized sfHMQC cross-peak intensities (*I*/*I*_o_) as a function of residue number for 60 μM Ac-αSyn in the presence of 60 μM Cu(ii) and increasing concentrations Gly rHSA (yellow). (h–i) Isolated *I*/*I*_o_ profiles for residues His-50 and Asp-121 plotted against increasing concentrations of Gly rHSA (yellow), and rHSA as a reference (gray dashed lines). Experimental points were fitted to a Hill-like model. Error bars represent the standard deviation of three neighboring residues. (j) Overlaid contour plots of the ^1^H–^15^N sfHMQC spectra of 60 μM aSyn (black) *vs.* Ac-αSyn in the presence of 60 μM Cu(ii) and 15 μM rHSA (grey). (k) as (j) but using 15 μM of Gly rHSA. Spectra were acquired at 10 °C in 50 mM HEPES, pH 7.4.

Incubation of HSA with MGO causes the MW of HSA to increase as shown by MALDI-TOF mass spectrometry (MS; Fig. 4a and b), in agreement with previous reports on MGO-induced HSA glycation.^78,86^ The mass-shift observed by MS (Fig. 4a and b) reflects the formation of advanced MGO-induced glycation end products (AGEs), which involve primarily arginine and lysine residues (Fig. 4c). Overall, our MS data (Fig. 4a and b) indicate that MGO-incubation produced a mass shift on HSA of 585 Da which can be explained by the reaction of MGO (72 g mol^−1^) with approximately 10–11 arginine and/or lysine residues, considering the elimination of water upon the reaction of these amino acids with MGO. This estimate is in full agreement with previous reports indicating an average molecular weight increase of 53.9 Da per reacted MGO (Fig. 4c).^86^

**Fig. 5:**
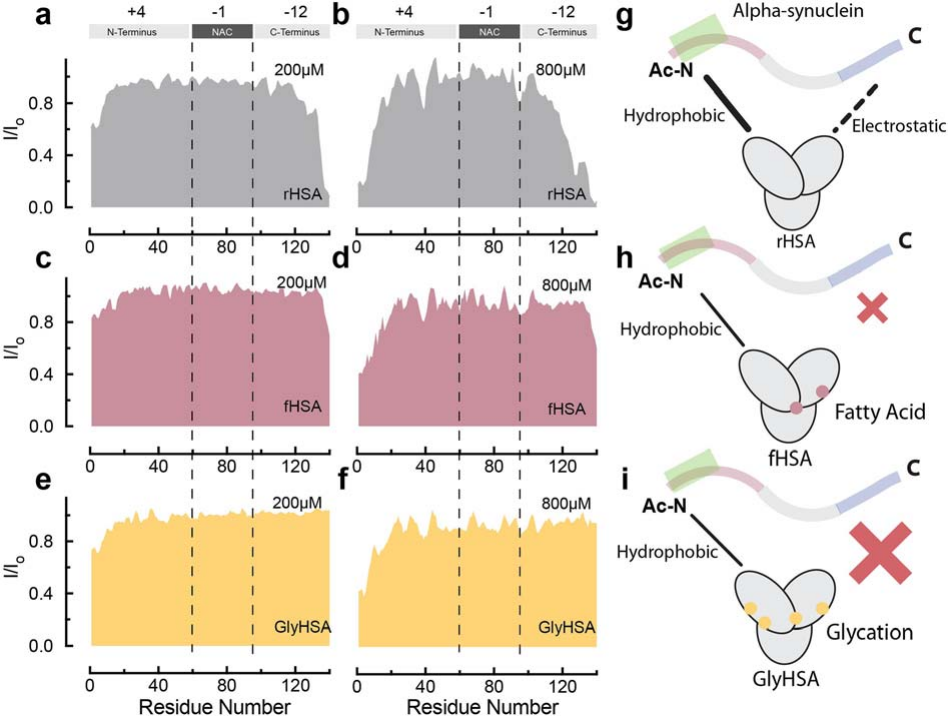
Binding of long chain fatty acids and glycation decrease binding of HSA to Ac-αSyn. (a–f) Normalized sfHMQC cross-peak intensities (*I*/*I*_o_) as a function of residue number for the backbone amide groups of 60 μM Ac-αSyn in the presence of 200 μM (a) and 800 μM rHSA (b), 200 μM (c) and 800 μM fHSA (d), 200 μM (e) and 800 μM GlyHSA (f). (g–i) Cartoon representation of HSA interactions with Ac-αSyn in the absence of modifications (g), in the presence of fatty acids (h) and after glycation (i). Spectra were acquired at 10 °C in 50 mM HEPES, pH 7.4.

Increasing concentrations of GlyHSA were titrated into Cu(ii)-bound Ac-αSyn and the titration was monitored through sfHMQC NMR experiments (Fig. 4d–g), similar to rHSA and fHSA. Our data show that the sequestration of Cu(ii) from Ac-αSyn was impaired (Fig. 4j and k), as the *I*/*I*_o_ recovery curves for both His-50 and Asp-121 are consistently shifted to higher albumin concentrations (Fig. 4h and i). Interestingly, the sigmoidal shape of the curves was largely preserved, suggesting that, although the affinity of GlyHSA for Cu(ii) ions decreases, the cooperativity between the metal HSA binding sites for Cu(ii) is still largely preserved (Fig. 4h, i and S4†). Similar to the non-glycated rHSA, Fig. S4† shows that even after rescaling the GlyHSA concentration we cannot recapitulate the chelation profile of EDTA. Interestingly, this is in stark contrast to fHSA, where the binding of ∼one-two LCFAs affects the cooperativity between the metal binding sites of HSA. Previously it was shown that MGO-glycated albumin exhibits a reduced coordination of Cu(ii), which can be attributed to the glycation of the NTS.^87^ In our case, this could explain the shift of the curves to the right (Fig. 4h and i), which corresponds to a decreased Cu(ii) affinity. In contrast, the binding of LCFAs leads to an allosteric effect on HSA, as discussed above, that disrupts the cooperativity between its metal binding sites. However, these experiments don't address how glycation and LCFAs affect the binding of HSA to Ac-αSyn. To this end, we acquired spectra of Ac-αSyn in the presence of different rHSA, fHSA and GlyHSA concentrations (Fig. 5).

### LCFA-binding and glycation silence the interactions between albumin and the CTR of Ac-αSyn

At physiological relevant plasma concentrations, HSA binds αSyn at both the N- and C-termini.^46^ The interaction at the C-termini is primarily electrostatically driven and is weakened by the binding of fatty acids to HSA. However, the results pertain to non-acetylated αSyn, while aN-terminally acetylated αSyn (Ac-αSyn) is the relevant physiological form^88^ and the one used for all our experiments here. Hence, we tested how αSyn's N-terminal acetylation, as well as glycation and fatty acid-binding to HSA, affect the interaction between both proteins.

The sfHMQC intensity losses observed for Ac-αSyn in the presence of rHSA and fHSA (Fig. 5a–d) revealed that αSyn's N-terminal acetylation has a negligible effect on the previously described patterns of interactions between albumin and αSyn,^46^ with long-chain fatty acids preserving their ability to partially silence the interactions with the C-terminal region (Fig. 5g and h). Moreover, for GlyHSA we found that albumin's binding to the C-terminus of Ac-αSyn is completely abolished, with the intensity of all C-terminal residues unaffected upon the addition of GlyHSA (Fig. 5e, f and i). A viable explanation for this observation is that glycation affects positively charged arginine and lysine HSA residues, reducing their charge and hindering their ability to bind the negatively charged C-terminal region of Ac-αSyn. However, we still don't know how metals such as Cu(ii) affect the binding of albumin to Ac-αSyn.

### Cu(ii) and Zn(ii)-binding to HSA results in pervasively enhanced interactions of albumin with monomeric Ac-αSyn

**Fig. 6:**
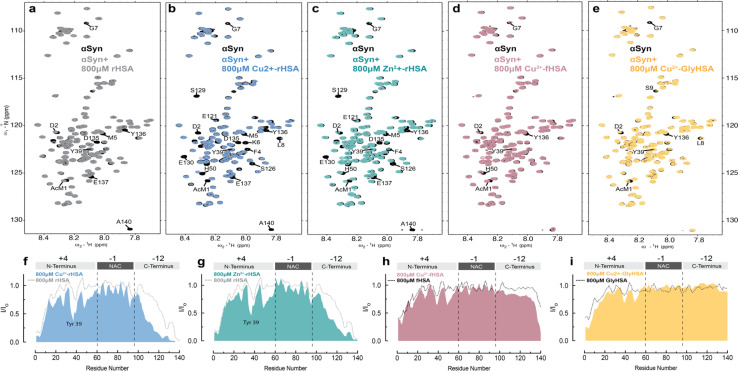
Metal binding to HSA enhances interactions between HSA and monomeric aSyn. (a–e) Overlay of the 2D-^15^N–^1^H sfHMQC spectra of 60 μM αSyn (black) and in the presence of 800 μM of rHSA (grey, a), 800 μM Cu(ii)-rHSA (blue, b), 800 μM Zn(ii)-rHSA (green, c), 800 μM Cu(ii)-fHSA (pink, d), 800 μM Cu(ii)-GlyHSA (yellow, e). (f–i) Normalized sfHMQC cross-peak intensities (*I*/*I*_o_) as a function of residue number for the backbone amide groups of 60 μM αSyn in the presence of 800 μM Cu(ii)-HSA (f), 800 μM Zn(ii)-rHSA (g), 800 μM Cu(ii)-fHSA (h), 800 μM Cu(ii)-GlyHSA (i), each compared to the corresponding profiles observed in the absence of added metals. Spectra were acquired at 10 °C in 50 mM HEPES, pH 7.4.

To probe how HSA binding of metal ions, such as Cu(ii), affects HSA's interactions with Ac-αSyn, we acquired sfHMQC spectra of ^15^N-labeled Ac-αSyn in the presence of plasma-like concentrations of unlabeled rHSA complexed with Cu(ii) ions (∼0.8 mM). To our surprise, we found that Cu(ii)-binding to HSA causes more pronounced sfHMQC intensity losses in the αSyn C-terminal region (Fig. 6a, b, and f). Additionally, we also observed enhanced intensity losses in the NTR region centered at Tyr39 (Fig. 6f), which together with the interaction at the N-terminus, has been reported as a canonical chaperone-αSyn binding site.^89^ To test whether this signal reduction was simply due to an inter-molecular paramagnetic effect of HSA-bound Cu(ii) ions, we repeated these experiments with Zn(ii) ions, which are diamagnetic and serve as a positive control. In addition, Zn(ii) and Cu(ii) ions, share the MBS and possibly site B in HSA^66^ and His 50 and Asp 121 in Ac-αSyn^90^ (Fig. 1a and b).

Similar to Cu(ii), complexation of HSA with Zn(ii) ions makes the albumin-induced sfHMQC intensity losses in Ac-αSyn more pronounced (Fig. 6c and g), ruling out that the sfHMQC intensity changes observed for Cu(ii)-bound HSA (Fig. 6f) are due primarily to inter-molecular paramagnetic relaxation enhancements. Furthermore, we did not observe any significant chemical shift difference between Ac-αSyn in the presence of unbound-HSA or Zn(ii)-bound HSA (Fig, S5a, b and d†), excluding the possibility of Zn(ii) ions being released by rHSA to bind αSyn. Taken together, these results indicate that when rHSA is complexed with metal ions, its canonical interactions with the Ac-αSyn CTR are enhanced and additional HSA-contact sites are observed in Ac-αSyn's NTR (Fig. 6f and g). These conclusions are in overall agreement with a previous report indicating that the binding of Zn(ii) ions to HSA enhances the chaperone binding to αSyn.^91^ However, previously it was found that Zn(ii)-bound HSA elicits an increased interaction with αSyn only at the N-terminal and NAC region, while the interactions with the C-terminal region are silenced,^91^ most likely because the previous experiments were conducted under different conditions, *i.e.* different salt concentrations, non-acetylated αSyn,^91^ 37 °C, and with different αSynZn(ii)-HSA ratios, which can account for the differences observed compared to our results. In addition, upon binding long-chain fatty acids or glycation, the pervasive interactions of metal-bound HSA are partially silenced (Fig. 6d, e, h and i), in line with what is observed in the absence of HSA-bound metals (Fig. 6h and i).

## Conclusions

**Fig. 7:**
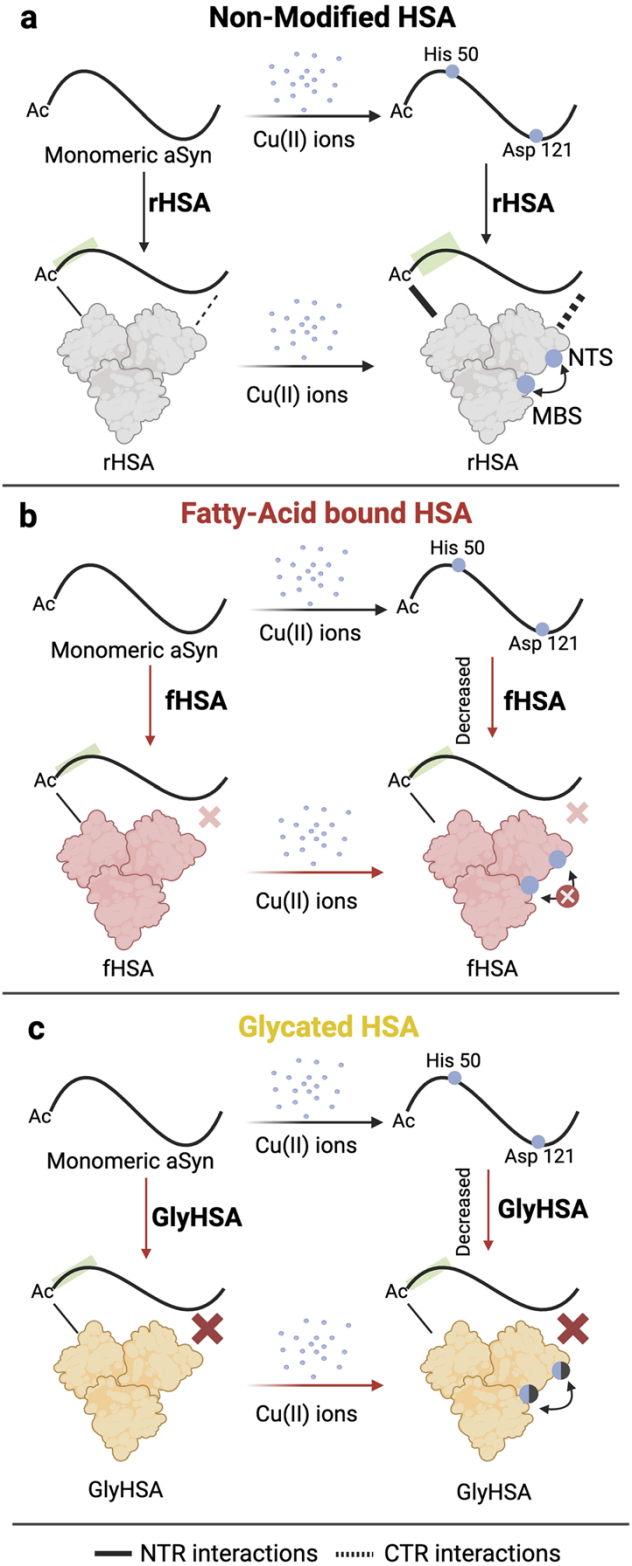
Mechanism of Cu(ii) transfer from Ac-αSyn to HSA and the implications of glycation and fatty acid binding to HSA. (a) Ac-αSyn binds Cu(ii) at two different sites the His-50 and Asp-121. Upon addition of de-fatted HSA (rHSA), Cu(ii) ions are chelated away from Ac-αSyn in a cooperative manner to the NTS and MBS in HSA, indicated by a black double headed arrow close to HSA. However, we cannot rule out potential contributions from site B in HSA. At the same time, binding of Cu(ii) ions to HSA increases the affinity of the chaperone for Ac-αSyn at the NTR and CTR. (b) Upon binding of LCFAs to HSA, the cooperativity between the NTS and MBS of HSA is compromised, which results in a decreased chelating ability of HSA. Additionally, unlike rHSA, Cu(ii) binding to fHSA does not significantly enhance the chaperone binding to Ac-αSyn. (c) Glycation of HSA decreases the binding of Cu(ii) ions (shaded blue spheres) but does not appreciably disrupt the cooperativity between the NTS and MBS sites of HSA. Addition of metal ions to GlyHSA does not significantly increase its binding to Ac-αSyn.

Our results paint a clear picture of the transfer of Cu(ii) from Ac-αSyn to HSA. Such transfer is relevant at CSF physiological conditions where HSA is responsible for avoiding aberrant interactions of toxic metal ions, such as copper, with αSyn and other disease-related IDPs. We found that the sequestration of Cu(ii) ions from Ac-αSyn by HSA follows a cooperative chelation mechanism, most likely involving its two primary Cu(ii) binding sites, NTS and MBS (Fig. 7a). Upon binding of LCFAs to HSA, the cooperative nature of Cu(ii) chelation is decreased. This leads to an overall reduced transfer of Cu(ii) ions from Ac-αSyn to fHSA compared to de-fatted HSA (Fig. 7b). Our work also considered posttranslational modifications of HSA, such as glycation. Addition of the glycating agent MGO decreases the binding affinity of Cu(ii) for HSA. However, unlike LCFA-binding, glycation does not appreciably perturb the cooperativity between the NTS and MBS (Fig. 7b).

We also investigated the interactions of monomeric Ac-αSyn with HSA in the presence of metal ions, fatty acids, and glycation. To our surprise, when the chaperone is bound to Cu(ii) and Zn(ii) ions, it displays increased binding at the NTR, and CTR of Ac-αSyn (Fig. 7a). These metal-enhanced interactions are weakened or largely silenced when HSA is bound to fatty acids or glycated, respectively (Fig. 7b and c). Furthermore, the previously reported interactions of non-acetylated αSyn with HSA in the presence or absence of fatty acids are conserved even when αSyn is acetylated. Overall, our study not only emphasizes the importance of fatty acid binding and age-related posttranslational modifications such as glycation for the neuroprotective mechanisms of HSA, but also highlights the potential of αSyn as a viable NMR-based sensor to investigate HSA-metal ions interactions. The concepts presented here are also relevant to understand the mechanism of action of albumin as a biotherapeutic^92–94^ for neurodegeneration.

## Experimental section

### Alpha synuclein expression and purification

*Escherichia coli* BL21 (DE3) cells and the pT7-7 plasmid were used to express αSyn, as previously described.^46^ N-terminally acetylated (Ac) αSyn was obtained through the co-transformation of *E. coli* with pT7-7 and a second plasmid (pACYC) encoding for the *Schizosaccharomyzes pombe* NatB acetyltransferase complex.^95^ The two plasmids exhibit distinctive antibiotic resistance, specifically for ampicillin and chloramphenicol, to select double-transformed *E. coli* colonies. Briefly, as before,^46^ bacteria were grown at 37 °C with 50 μg mL^−1^ of ampicillin and 25 μg mL^−1^ chloramphenicol in ^15^N-ammonium chloride-enriched M9 minimal media. Upon ∼0.6–0.8 OD_600_, 100 μM of isopropyl β-d-1-thiogalactopyranoside (IPTG) was used to overexpress alpha-synuclein at 37 °C for 4 h. The cells were pelleted at 10 000 g for 10 min and stored at −80 °C until purification. For the purification of Ac-αSyn, the cell pellets were dissolved in lysis buffer (10 mM Tris–HCl pH 8, 1 mM EDTA, 1 mM AEBSF protease inhibitor) and lysed by three cycles of freeze-thawing followed by sonication. After sonication, the cell lysate was boiled for 20 min and separated by centrifugation at 19 500 g for 1 h. Afterwards, the supernatant was treated with streptomycin sulphate to a final concentration of 10 mg mL^−1^. The mixture was stirred for 20 min at 4 °C and the precipitate was removed by centrifugation at 19 500 g for 10 min. Subsequently, 360 mg mL^−1^ of ammonium sulphate was used to precipitate the protein. The solution was mixed for 1 h at 4 °C and centrifuged at 9500 g for 15 min. The protein precipitate (pellet) was resuspended in 25 mM Tris–HCl, pH 7.7 (Buffer A), and loaded onto a size exclusion chromatography (SEC) (HiLoad 16/60 Superdex 200 increase gel filtration column, GE Healthcare) equilibrated with buffer A. Next, the fractions containing monomeric alpha-synuclein were injected onto an anion exchange column (HiTrap Q Sepharose high performance, GE Healthcare) and eluted with a 0–600 mM NaCl (Buffer B) step gradient. At ∼50% NaCl the fractions containing purified alpha-synuclein were further loaded onto a SEC (HiLoad 16/60 Superdex 200 increase gel filtration column, GE Healthcare) equilibrated with double-distilled H_2_O to extract monomeric Ac-αSyn and discard any oligomers formed during the purification. The fractions with monomeric alpha-synuclein were lyophilized and stored at −20 °C. Protein concentration was determined by UV absorbance at 280 nm and *E* = 5600 M^−1^ cm^−1^ using a NanoDrop One^c^ (Thermo Fisher).

### NMR sample preparation

Monomeric Ac-αSyn was prepared by dissolving the lyophilized powder in 50 mM HEPES pH 7.4 with 5% D_2_O for NMR experiments. Fresh samples were instantaneously analyzed to avoid Ac-αSyn aggregation.

### Preparation of other stock solutions

Lyophilized fatty acid and globulin-free human serum albumin (rHSA; Sigma-Aldrich A3782) and globulin-free human serum albumin (fHSA; Sigma-Aldrich A8763) were dissolved in 50 mM HEPES pH 7.4 or PBS pH 7.4 buffers. Copper(ii) chloride (99.9% trace metals basis) was purchased from Sigma (CAS number: 7447-39-4). A stock solution of 0.1 M was prepared using filtered double-distilled H_2_O treated with Chelex 100 resin (Bio-Rad 1422822).

### Preparation and characterization of glycated human serum albumin

Glycation of commercially available fatty acid and globulin-free HSA (rHSA) was conducted as previously described^86^ by incubating 40 mg mL^−1^ of HSA in 1 × PBS with 10 mM of methyl glyoxal (Sigma-Aldrich CAS:78-98-8) at 37 °C for 48 h. The product of this incubation (Gly-HSA) was dialyzed against 1 × PBS to remove the excess of methyl glyoxal. GlyHSA was lyophilized and stored at −20 °C. Control non-glycated HSA was treated identically to GlyHSA (*i.e.*, incubation at 37 °C for 48 h) except that no methyl glyoxal was present in the reaction buffer. The glycation stage of GlyHSA was analyzed by MALDI TOF-MS as described previously.^81^

### Solution NMR experiments

All NMR spectra were acquired on a Bruker 700 Advance or NEO spectrometer equipped with a TCI cryoprobe. The spectra were analyzed with TopSpin 4.0.7 and NMRFAM Sparky.

#### ^1^H–^15^N HSQC and SO-FAST HMQC intensity analysis to monitor Cu(ii) and HSA binding to Ac-αSyn

HSA and Cu(ii) binding to Ac-αSyn was studied by losses in ^1^H–^15^N HSQC or SO-FAST HMQC intensities upon the addition of HSA and Cu(ii) compared to a sample of Ac-αSyn alone. ^1^H–^15^N HSQC spectra were recorded at 283 K, with a recycle delay of 1.0 s, 16 scans, and 4 K (*t*_2_) and 300 (*t*_1_) complex points and spectral widths of 14.05 ppm (^1^H) and 31.82 ppm (^15^N). For SO-FAST HMQC experiments the temperature was also 283 K with a recycle delay of 0.5 s, 64 scans, and 2 K (*t*_2_) and 300 (*t*_1_) complex points for spectral widths of 16.22 ppm (^1^H) and 35 ppm (^15^N). Spectra were obtained for Ac-αSyn with and without rHSA, fHSA or Gly-HSA in the presence or absence of Cu(ii) ions.

#### Characterization of fatty acid bound HSA (fHSA) through CONFA

To estimate the amount of long-chain fatty acids (LCFAs) bound to commercially available HSA, we used ^13^C-methyl-labeled oleic acid for the NMR-based assessment of albumin-bound LCFA concentration (CONFA)^73^ approach. Lyophilized, commercially available fatty acid bound (globulin free) human serum albumin (fHSA; Sigma-Aldrich A8763) was dissolved in NMR buffer (50 mM Sodium phosphate, 50 mM NaCl, pH 7.4, 99% deuterium oxide) as previously described.^73^ fHSA concentration was checked using UV absorbance at 280 nm and *E* = 35 700 M^−1^ cm^−1^ using a NanoDrop One^c^ (Thermo Fisher). ^12^C-oleic acid was obtained from Sigma-Aldrich (O1008, ≥99% pure based on TLC and GC, CAS: 112-80-1), while ^13^C oleic acid was purchased from Cambridge Isotope Laboratories (CLM2492, 98% chemical purity and 99% isotopic enrichment). ^12^C and ^13^C oleic acid 100 mM stocks were prepared in 100% d_6_-dimethyl sulfoxide (DMSO) from Cambridge Isotope Laboratories. CONFA samples were prepared as previously described.^73^ Briefly, fatty acid stock solutions were preheated at 50 °C for 5 min and added to a 0.5 mM fHSA solution, which was preincubated at 37 °C in advance for 30 min. The mixture of fHSA and fatty acids was incubated at 37 °C for two more hours before acquiring 1D ^13^C NMR spectra. The 1D-^13^C spectra were recorded at 298 K with ^1^H decoupling and a spectral width of 41 666.66 Hz and 65k points. The recycle delay was 1 s, and the number of scans was 4 K, preceded by 32 dummy scans.

## Data availability

Supporting data is available upon request.

## Author contributions

K. M. P, R. A. and G. M. designed the experimental plan and research; K. M. P. and J. H. conducted experiments; K. M. P. analysed the data; K. M. P. and G. M. wrote the manuscript. All authors approved the final version of this paper.

## Conflicts of interest

There is no conflict of interest to declare.

## Supplementary Material

SC-015-D3SC06285F-s001

## References

[cit1] Benskey M. J., Perez R. G., Manfredsson F. P. (2016). The Contribution of Alpha Synuclein to Neuronal Survival and Function – Implications for Parkinson's Disease. J. Neurochem..

[cit2] Emamzadeh F. N. (2016). Alpha-Synuclein Structure, Functions, and Interactions. J. Res. Med. Sci..

[cit3] Luth E. S., Stavrovskaya I. G., Bartels T., Kristal B. S., Selkoe D. J. (2014). Soluble, Prefibrillar α-Synuclein Oligomers Promote Complex I-Dependent, Ca2+-Induced Mitochondrial Dysfunction. J. Biol. Chem..

[cit4] Rodriguez J. A., Ivanova M. I., Sawaya M. R., Cascio D., Reyes F. E., Shi D., Sangwan S., Guenther E. L., Johnson L. M., Zhang M., Jiang L., Arbing M. A., Nannenga B. L., Hattne J., Whitelegge J., Brewster A. S., Messerschmidt M., Boutet S., Sauter N. K., Gonen T., Eisenberg D. S. (2015). Structure of the Toxic Core of α-Synuclein from Invisible Crystals. Nature.

[cit5] Winner B., Jappelli R., Maji S. K., Desplats P. A., Boyer L., Aigner S., Hetzer C., Loher T., Vilar M., Campioni S., Tzitzilonis C., Soragni A., Jessberger S., Mira H., Consiglio A., Pham E., Masliah E., Gage F. H., Riek R. (2011). In Vivo Demonstration That α-Synuclein Oligomers Are Toxic. Proc. Natl. Acad. Sci. U. S. A..

[cit6] Grey M., Linse S., Nilsson H., Brundin P., Sparr E. (2011). Membrane Interaction of α-Synuclein in Different Aggregation States. J. Parkinson's Dis..

[cit7] van Rooijen B. D., Claessens M. M., Subramaniam V. (2010). Membrane Interactions of Oligomeric Alpha-Synuclein: Potential Role in Parkinsons Disease. Curr. Protein Pept. Sci..

[cit8] Danzer K. M., Haasen D., Karow A. R., Moussaud S., Habeck M., Giese A., Kretzschmar H., Hengerer B., Kostka M. (2007). Different Species of α-Synuclein Oligomers Induce Calcium Influx and Seeding. J. Neurosci..

[cit9] Fusco G., Chen S. W., Williamson P. T. F., Cascella R., Perni M., Jarvis J. A., Cecchi C., Vendruscolo M., Chiti F., Cremades N., Ying L., Dobson C. M., De Simone A. (2017). Structural Basis of Membrane Disruption and Cellular Toxicity by A-Synuclein Oligomers. Science.

[cit10] Ahmed R., Melacini G. (2021). Biophysical Toolset to Probe the Microscopic Processes Underlying Protein Aggregation and Its Inhibition by Molecular Chaperones. Biophys. Chem..

[cit11] Bhattacharyya D., Mohite G. M., Krishnamoorthy J., Gayen N., Mehra S., Navalkar A., Kotler S. A., Ratha B. N., Ghosh A., Kumar R., Garai K., Mandal A. K., Maji S. K., Bhunia A. (2019). Lipopolysaccharide from Gut Microbiota Modulates α-Synuclein Aggregation and Alters Its Biological Function. ACS Chem. Neurosci..

[cit12] Bhattacharyya D., Kumar R., Mehra S., Ghosh A., Maji S. K., Bhunia A. (2018). Multitude NMR Studies of α-Synuclein Familial Mutants: Probing Their Differential Aggregation Propensities. Chem. Commun..

[cit13] Stephens A. D., Zacharopoulou M., Kaminski Schierle G. S. (2019). The Cellular Environment Affects Monomeric α-Synuclein Structure. Trends Biochem. Sci..

[cit14] Binolfi A., Rasia R. M., Bertoncini C. W., Ceolin M., Zweckstetter M., Griesinger C., Jovin T. M., Fernández C. O. (2006). Interaction of α-Synuclein with Divalent Metal Ions Reveals Key Differences: A Link between Structure, Binding Specificity and Fibrillation Enhancement. J. Am. Chem. Soc..

[cit15] Runfola M., De Simone A., Vendruscolo M., Dobson C. M., Fusco G. (2020). The N-Terminal Acetylation of α-Synuclein Changes the Affinity for Lipid Membranes but Not the Structural Properties of the Bound State. Sci. Rep..

[cit16] Fusco G., De Simone A., Arosio P., Vendruscolo M., Veglia G., Dobson C. M. (2016). Structural Ensembles of Membrane-Bound α-Synuclein Reveal the Molecular Determinants of Synaptic Vesicle Affinity. Sci. Rep..

[cit17] Masuda M., Suzuki N., Taniguchi S., Oikawa T., Nonaka T., Iwatsubo T., Hisanaga S. I., Goedert M., Hasegawa M. (2006). Small Molecule Inhibitors of α-Synuclein Filament Assembly. Biochemistry.

[cit18] Lautenschläger J., Stephens A. D., Fusco G., Ströhl F., Curry N., Zacharopoulou M., Michel C. H., Laine R., Nespovitaya N., Fantham M., Pinotsi D., Zago W., Fraser P., Tandon A., St George-Hyslop P., Rees E., Phillips J. J., De Simone A., Kaminski C. F., Schierle G. S. K. (2018). C-Terminal Calcium Binding of α-Synuclein Modulates Synaptic Vesicle Interaction. Nat. Commun..

[cit19] Binolfi A., Quintanar L., Bertoncini C. W., Griesinger C., Fernández C. O. (2012). Bioinorganic Chemistry of Copper Coordination to Alpha-Synuclein: Relevance to Parkinson's Disease. Coord. Chem. Rev..

[cit20] Rasia R. M., Bertoncini C. W., Marsh D., Hoyer W., Cherny D., Zweckstetter M., Griesinger C., Jovin T. M., Fernández C. O. (2005). Structural Characterization of Copper(II) Binding to α-Synuclein: Insights into the Bioinorganic Chemistry of Parkinson's Disease. Proc. Natl. Acad. Sci. U. S. A..

[cit21] Okita Y., Rcom-H’cheo-Gauthier A. N., Goulding M., Chung R. S., Faller P., Pountney D. L. (2017). Metallothionein, Copper and Alpha-Synuclein in Alpha-Synucleinopathies. Front. Neurosci..

[cit22] Miotto M. C., Rodriguez E. E., Valiente-Gabioud A. A., Torres-Monserrat V., Binolfi A., Quintanar L., Zweckstetter M., Griesinger C., Fernández C. O. (2014). Site-Specific Copper-Catalyzed Oxidation of α-Synuclein: Tightening the Link between Metal Binding and Protein Oxidative Damage in Parkinson's Disease. Inorg. Chem..

[cit23] Mansueto S., Fusco G., De Simone A. (2023). α-Synuclein and Biological Membranes: The Danger of Loving Too Much. Chem. Commun..

[cit24] Gonzalez-Garcia M., Fusco G., De Simone A. (2023). Metal Interactions of α-Synuclein Probed by NMR Amide-Proton Exchange. Front. Chem..

[cit25] González N., Arcos-López T., König A., Quintanar L., Menacho Márquez M., Outeiro T. F., Fernández C. O. (2019). Effects of Alpha-Synuclein Post-Translational Modifications on Metal Binding. J. Neurochem..

[cit26] González N., Arcos-López T., König A., Quintanar L., Menacho Márquez M., Outeiro T. F., Fernández C. O. (2019). Effects of Alpha-Synuclein Post-Translational Modifications on Metal Binding. J. Neurochem..

[cit27] Chakraborty R., Dey S., Sil P., Paul S. S., Bhattacharyya D., Bhunia A., Sengupta J., Chattopadhyay K. (2021). Conformational Distortion in a Fibril-Forming Oligomer Arrests Alpha-Synuclein Fibrillation and Minimizes Its Toxic Effects. Commun. Biol..

[cit28] El-Agnaf O. M. A., Salem S. A., Paleologou K. E., Cooper L. J., Fullwood N. J., Gibson M. J., Curran M. D., Court J. A., Mann D. M. A., Ikeda S.-I., Cookson M. R., Hardy J., Allsop D. (2003). α-Synuclein Implicated in Parkinson's Disease Is Present in Extracellular Biological Fluids, Including Human Plasma. FASEB J..

[cit29] Lee H.-J., Bae E.-J., Lee S.-J. (2014). Extracellular α-Synuclein—a Novel and Crucial Factor in Lewy Body Diseases. Nat. Rev. Neurol..

[cit30] Ma J., Gao J., Wang J., Xie A. (2019). Prion-like Mechanisms in Parkinson's Disease. Front. Neurosci..

[cit31] Finn T. E., Nunez A. C., Sunde M., Easterbrook-Smith S. B. (2012). Serum Albumin Prevents Protein Aggregation and Amyloid Formation and Retains Chaperone-like Activity in the Presence of Physiological Ligands. J. Biol. Chem..

[cit32] Ahmed R., Melacini G. (2018). A Solution NMR Toolset to Probe the Molecular Mechanisms of Amyloid Inhibitors. Chem. Commun..

[cit33] Bellomo G., Bologna S., Cerofolini L., Paciotti S., Gatticchi L., Ravera E., Parnetti L., Fragai M., Luchinat C. (2019). Dissecting the Interactions between Human Serum Albumin and α-Synuclein: New Insights on the Factors Influencing α-Synuclein Aggregation in Biological Fluids. J. Phys. Chem. B.

[cit34] Theillet F.-X., Binolfi A., Bekei B., Martorana A., Rose H. M., Stuiver M., Verzini S., Lorenz D., van Rossum M., Goldfarb D., Selenko P. (2016). Structural Disorder of Monomeric α-Synuclein Persists in Mammalian Cells. Nature.

[cit35] Kakinen A., Javed I., Faridi A., Davis T. P., Ke P. C. (2018). Serum Albumin Impedes the Amyloid Aggregation and Hemolysis of Human Islet Amyloid Polypeptide and Alpha Synuclein. Biochim. Biophys. Acta, Biomembr..

[cit36] Ahmed R., Huang J., Akimoto M., Shi T., Melacini G. (2021). Atomic Resolution Map of Hierarchical Self-Assembly for an Amyloidogenic Protein Probed through Thermal 15N-R2Correlation Matrices. J. Am. Chem. Soc..

[cit37] Milojevic J., Raditsis A., Melacini G. (2009). Human Serum Albumin Inhibits Abeta Fibrillization through a “Monomer-Competitor” Mechanism. Biophys. J..

[cit38] Milojevic J., Esposito V., Das R., Melacini G. (2007). Understanding the Molecular Basis for the Inhibition of the Alzheimer's Abeta-Peptide Oligomerization by Human Serum Albumin Using Saturation Transfer Difference and off-Resonance Relaxation NMR Spectroscopy. J. Am. Chem. Soc..

[cit39] Milojevic J., Melacini G. (2011). Stoichiometry and Affinity of the Human Serum Albumin-Alzheimer’s Aβ Peptide Interactions. Biophys. J..

[cit40] Algamal M., Milojevic J., Jafari N., Zhang W., Melacini G. (2013). Mapping the Interactions between the Alzheimer's Aβ-Peptide and Human Serum Albumin beyond Domain Resolution. Biophys. J..

[cit41] Algamal M., Ahmed R., Jafari N., Ahsan B., Ortega J., Melacini G. (2017). Atomic-Resolution Map of the Interactions between an Amyloid Inhibitor Protein and Amyloid β (Aβ) Peptides in the Monomer and Protofibril States. J. Biol. Chem..

[cit42] Choi T. S., Lee H. J., Han J. Y., Lim M. H., Kim H. I. (2017). Molecular Insights into Human Serum Albumin as a Receptor of Amyloid-β in the Extracellular Region. J. Am. Chem. Soc..

[cit43] Korshavn K. J., Satriano C., Lin Y., Zhang R., Dulchavsky M., Bhunia A., Ivanova M. I., Lee Y. H., La Rosa C., Lim M. H., Ramamoorthy A. (2017). Reduced Lipid Bilayer Thickness Regulates the Aggregation and Cytotoxicity of Amyloid-β. J. Biol. Chem..

[cit44] Pagano K., Tomaselli S., Molinari H., Ragona L. (2020). Natural Compounds as Inhibitors of Aβ Peptide Aggregation: Chemical Requirements and Molecular Mechanisms. Front. Neurosci..

[cit45] Tomaselli S., La Vitola P., Pagano K., Brandi E., Santamaria G., Galante D., D'Arrigo C., Moni L., Lambruschini C., Banfi L., Lucchetti J., Fracasso C., Molinari H., Forloni G., Balducci C., Ragona L. (2019). Biophysical and in Vivo Studies Identify a New Natural-Based Polyphenol, Counteracting Aβ Oligomerization in Vitro and Aβ Oligomer-Mediated Memory Impairment and Neuroinflammation in an Acute Mouse Model of Alzheimer's Disease. ACS Chem. Neurosci..

[cit46] Ahmed R., Huang J., Weber D. K., Gopinath T., Veglia G., Akimoto M., Khondker A., Rheinstädter M. C., Huynh V., Wylie R. G., Bozelli J. C., Epand R. M., Melacini G. (2020). Molecular Mechanism for the Suppression of Alpha Synuclein Membrane Toxicity by an Unconventional Extracellular Chaperone. J. Am. Chem. Soc..

[cit47] Lee H. J., Korshavn K. J., Kochi A., Derrick J. S., Lim M. H. (2014). Cholesterol and Metal Ions in Alzheimer's Disease. Chem. Soc. Rev..

[cit48] Leal S. S., Botelho H. M., Gomes C. M. (2012). Metal Ions as Modulators of Protein Conformation and Misfolding in Neurodegeneration. Coord. Chem. Rev..

[cit49] Parthasarathy S., Long F., Miller Y., Xiao Y., McElheny D., Thurber K., Ma B., Nussinov R., Ishii Y. (2011). Molecular-Level Examination of Cu2+ Binding Structure for Amyloid Fibrils of 40-Residue Alzheimer's β by Solid-State NMR Spectroscopy. J. Am. Chem. Soc..

[cit50] Miller Y., Ma B., Nussinov R. (2010). Zinc Ions Promote Alzheimer Aβ Aggregation via Population Shift of Polymorphic States. Proc. Natl. Acad. Sci. U. S. A..

[cit51] Kim K. S., Choi S. Y., Kwon H. Y., Won M. H., Kang T.-C., Kang J. H. (2002). Aggregation of α-Synuclein Induced by the Cu,Zn-Superoxide Dismutase and Hydrogen Peroxide System. Free Radical Biol. Med..

[cit52] Faller P. (2009). Copper and Zinc Binding to Amyloid-β: Coordination, Dynamics, Aggregation, Reactivity and Metal-Ion Transfer. ChemBioChem.

[cit53] Kim A. C., Lim S., Kim Y. K. (2018). Metal Ion Effects on Aβ and Tau Aggregation. Int. J. Mol. Sci..

[cit54] Breydo L., Uversky V. N. (2011). Role of Metal Ions in Aggregation of Intrinsically Disordered Proteins in Neurodegenerative Diseases. Metallomics.

[cit55] del Barrio M. , Borghesani V., Hureau C. and Faller P., in Chapter 14 – Metal-Binding to Amyloid-β Peptide: Coordination, Aggregation, and Reactive Oxygen Species Production, Biometals in Neurodegenerative Diseases, Mechanisms and Therapeutics, ed. A. White, M. Aschner, L. Costa and A. Bush, Academic Press, Elsevier, 2017, 1st edn, pp. 265–281, 10.1016/B978-0-12-804562-6.00014-2

[cit56] Faller P., Hureau C., La Penna G. (2014). Metal Ions and Intrinsically Disordered Proteins and Peptides: From Cu/Zn Amyloid-β to General Principles. Acc. Chem. Res..

[cit57] Ahmadi S., Zhu S., Sharma R., Wilson D. J., Kraatz H.-B. (2019). Interaction of Metal Ions with Tau Protein. The Case for a Metal-Mediated Tau Aggregation. J. Inorg. Biochem..

[cit58] Perrone L., Mothes E., Vignes M., Mockel A., Figueroa C., Miquel M. C., Maddelein M. L., Faller P. (2010). Copper Transfer from Cu-Aβ to Human Serum Albumin Inhibits Aggregation, Radical Production and Reduces Aβ Toxicity. ChemBioChem.

[cit59] Appleton D. W., Sarkar B. (1971). The Absence of Specific Copper(II)-Binding Site in Dog Albumin. J. Biol. Chem..

[cit60] Bal W., Christodoulou J., Sadler P. J., Tucker A. (1998). Multi-Metal Binding Site of Serum Albumin. J. Inorg. Biochem..

[cit61] Brutscher B. , SOFAST HMQC BT – Encyclopedia of Biophysics, ed. G. C. K. Roberts, Springer Berlin Heidelberg, Berlin, Heidelberg, 2013, p. 2407, 10.1007/978-3-642-16712-6_347

[cit62] Binolfi A., Rodriguez E. E., Valensin D., D'Amelio N., Ippoliti E., Obal G., Duran R., Magistrato A., Pritsch O., Zweckstetter M., Valensin G., Carloni P., Quintanar L., Griesinger C., Fernández C. O. (2010). Bioinorganic Chemistry of Parkinson's Disease: Structural Determinants for the Copper-Mediated Amyloid Formation of Alpha-Synuclein. Inorg. Chem..

[cit63] Ravera E., Takis P. G., Fragai M., Parigi G., Luchinat C. (2018). NMR Spectroscopy and Metal Ions in Life Sciences. Eur. J. Inorg. Chem..

[cit64] Aime S., Canton S., Crich S. G., Terreno E. (2002). 1H and 17O Relaxometric Investigations of the Binding of Mn(II) Ion to Human Serum Albumin. Magn. Reson. Chem..

[cit65] Fanali G., Cao Y., Ascenzi P., Fasano M. (2012). Mn(II) Binding to Human Serum Albumin: A ^1^H-NMR Relaxometric Study. J. Inorg. Biochem..

[cit66] Bal W., Sokołowska M., Kurowska E., Faller P. (2013). Binding of Transition Metal Ions to Albumin: Sites, Affinities and Rates. Biochim. Biophys. Acta, Gen. Subj..

[cit67] Rózga M., Bal W. (2010). The Cu(II)/Aβ/Human Serum Albumin Model of Control Mechanism for Copper-Related Amyloid Neurotoxicity. Chem. Res. Toxicol..

[cit68] Stanyon H. F., Viles J. H. (2012). Human Serum Albumin Can Regulate Amyloid-β Peptide Fiber Growth in the Brain Interstitium: Implications for Alzheimer Disease. J. Biol. Chem..

[cit69] Wang L., Hu W., Wang J., Fang F., Cheng G., Jiang Y., Xiao H., Wan Q. (2017). Impact of Serum Uric Acid, Albumin and Their Interaction on Parkinson's Disease. Neurol. Sci..

[cit70] Sun S., Wen Y., Li Y. (2022). Serum Albumin, Cognitive Function, Motor Impairment, and Survival Prognosis in Parkinson Disease. Medicine.

[cit71] Klonoff-Cohen H., Barrett-Connor E. L., Edelstein S. L. (1992). Albumin Levels as a Predictor of Mortality in the Healthy Elderly. J. Clin. Epidemiol..

[cit72] Fasano M., Curry S., Terreno E., Galliano M., Fanali G., Narciso P., Notari S., Ascenzi P. (2005). The Extraordinary Ligand Binding Properties of Human Serum Albumin. IUBMB Life.

[cit73] Jafari N., Ahmed R., Gloyd M., Bloomfield J., Britz-McKibbin P., Melacini G. (2016). Allosteric Sensing of Fatty Acid Binding by NMR: Application to Human Serum Albumin. J. Med. Chem..

[cit74] Krenzel E. S., Chen Z., Hamilton J. A. (2013). Erratum: Correspondence of Fatty Acid and Drug Binding Sites on Human Serum Albumin: A Two-Dimensional Nuclear Magnetic Resonance Study (Biochemistry (2013) 52:9 (1559-1567) DOI:10.1021/Bi301458b).. Biochemistry.

[cit75] Lu J., Stewart A. J., Sleep D., Sadler P. J., Pinheiro T. J. T., Blindauer C. A. (2012). A Molecular Mechanism for Modulating Plasma Zn Speciation by Fatty Acids. J. Am. Chem. Soc..

[cit76] Blindauer C. A., Khazaipoul S., Yu R., Stewart A. J. (2016). Fatty Acid-Mediated Inhibition of Metal Binding to the Multi-Metal Site on Serum Albumin: Implications for Cardiovascular Disease. Curr. Top. Med. Chem..

[cit77] Lu J., Stewart A. J., Sadler P. J., Pinheiro T. J. T., Blindauer C. A. (2012). Allosteric Inhibition of Cobalt Binding to Albumin by Fatty Acids: Implications for the Detection of Myocardial Ischemia. J. Med. Chem..

[cit78] Arasteh A., Farahi S., Habibi-Rezaei M., Moosavi-Movahedi A. A. (2014). Glycated Albumin: An Overview of the In Vitro Models of an In Vivo Potential Disease Marker. J. Diabetes Metab. Disord..

[cit79] Ahmed A., Shamsi A., Khan M. S., Husain F. M., Bano B. (2018). Methylglyoxal Induced Glycation and Aggregation of Human Serum Albumin: Biochemical and Biophysical Approach. Int. J. Biol. Macromol..

[cit80] Kannan S., Souchelnytskyi S. (2022). Review of Post-Translational Modification of Human Serum Albumin. Curr. Protein Pept. Sci..

[cit81] Paradela-Dobarro B., Rodiño-Janeiro B. K., Alonso J., Raposeiras-Roubín S., González-Peteiro M., González-Juanatey J. R., Álvarez E. (2015). Key Structural and Functional Differences between Early and Advanced Glycation Products. J. Mol. Endocrinol..

[cit82] Lee P., Wu X. (2015). Review: Modifications of Human Serum Albumin and Their Binding Effect. Curr. Pharm. Des..

[cit83] Maciążek-Jurczyk M., Szkudlarek A., Chudzik M., Pożycka J., Sułkowska A. (2018). Alteration of Human Serum Albumin Binding Properties Induced by Modifications: A Review. Spectrochim. Acta, Part A.

[cit84] Szkudlarek A., Maciązek-Jurczyk M., Chudzik M., Równicka-Zubik J., Sułkowska A. (2016). Alteration of Human Serum Albumin Tertiary Structure Induced by Glycation. Spectroscopic Study. Spectrochim. Acta, Part A.

[cit85] Iqbal S., Qais F. A., Alam M. M., Naseem I. (2018). Effect of Glycation on Human Serum Albumin-Zinc Interaction: A Biophysical Study. JBIC, J. Biol. Inorg. Chem..

[cit86] Soudahome A. G., Catan A., Giraud P., Kouao S. A., Guerin-Dubourg A., Debussche X., Le Moullec N., Bourdon E., Bravo S. B., Paradela-Dobarro B., Álvarez E., Meilhac O., Rondeau P., Couprie J. (2018). Glycation of Human Serum Albumin Impairs Binding to the Glucagon-like Peptide-1 Analogue Liraglutide. J. Biol. Chem..

[cit87] Corrales Escobosa A. R., Wrobel K., Yanez Barrientos E., Jaramillo Ortiz S., Ramirez Segovia A. S., Wrobel K. (2015). Effect of Different Glycation Agents on Cu(II) Binding to Human Serum Albumin, Studied by Liquid Chromatography, Nitrogen Microwave-Plasma Atomic-Emission Spectrometry, Inductively-Coupled-Plasma Mass Spectrometry, and High-Resolution Molecular-Mass Spect. Anal. Bioanal. Chem..

[cit88] Miotto M. C., Valiente-Gabioud A. A., Rossetti G., Zweckstetter M., Carloni P., Selenko P., Griesinger C., Binolfi A., Fernández C. O. (2015). Copper Binding to the N-Terminally Acetylated, Naturally Occurring Form of Alpha-Synuclein Induces Local Helical Folding. J. Am. Chem. Soc..

[cit89] Burmann B. M., Gerez J. A., Matečko-Burmann I., Campioni S., Kumari P., Ghosh D., Mazur A., Aspholm E. E., Šulskis D., Wawrzyniuk M., Bock T., Schmidt A., Rüdiger S. G. D., Riek R., Hiller S. (2020). Regulation of α-Synuclein by Chaperones in Mammalian Cells. Nature.

[cit90] Valiente-Gabioud A. A., Torres-Monserrat V., Molina-Rubino L., Binolfi A., Griesinger C., Fernández C. O. (2012). Structural Basis behind the Interaction of Zn2 + with the Protein α-Synuclein and the Aβ Peptide: A Comparative Analysis. J. Inorg. Biochem..

[cit91] Al-Harthi S., Kharchenko V., Mandal P., Gourdoupis S., Jaremko Ł. (2022). Zinc Ions Prevent α-Synuclein Aggregation by Enhancing Chaperone Function of Human Serum Albumin. Int. J. Biol. Macromol..

[cit92] Boada M., Martínez-Lage P., Serrano-Castro P., Costa M., Páez A. (2021). Therapeutic Plasma Exchange with Albumin: A New Approach to Treat Alzheimer's Disease. Expert Rev. Neurother..

[cit93] Boada M., López O. L., Olazarán J., Núñez L., Pfeffer M., Paricio M., Lorites J., Piñol-Ripoll G., Gámez J. E., Anaya F., Kiprov D., Lima J., Grifols C., Torres M., Costa M., Bozzo J., Szczepiorkowski Z. M., Hendrix S., Páez A. (2020). A Randomized, Controlled Clinical Trial of Plasma Exchange with Albumin Replacement for Alzheimer's Disease: Primary Results of the AMBAR Study. Alzheimers. Dement..

[cit94] Milojevic J., Costa M., Ortiz A. M., Jorquera J. I., Melacini G. (2014). In Vitro Amyloid-β Binding and Inhibition of Amyloid-β Self-Association by Therapeutic Albumin. J. Alzheimer's Dis..

[cit95] Bell R., Thrush R. J., Castellana-Cruz M., Oeller M., Staats R., Nene A., Flagmeier P., Xu C. K., Satapathy S., Galvagnion C., Wilson M. R., Dobson C. M., Kumita J. R., Vendruscolo M. (2022). N-Terminal Acetylation of α-Synuclein Slows down Its Aggregation Process and Alters the Morphology of the Resulting Aggregates. Biochemistry.

