# Supplementary material for: Inhibition of toxic metal-alpha synuclein interactions by human serum albumin
Source: Chem Sci. 2024 Jan 31;15(10):3502–15. doi: 10.1039/d3sc06285f (PMC10915811; doi:10.1039/d3sc06285f)
Supplement: SC-015-D3SC06285F-s001 [file SC-015-D3SC06285F-s001.pdf]

Electronic Supplementary Material (ESI) for Chemical Science.
This journal is © The Royal Society of Chemistry 2024

Inhibition of Toxic Metal-Alpha Synuclein Interactions by Human Serum Albumin

Karla Martinez Pomier,[a] Rashik Ahmed,[a] Jinfeng Huang[a] and Giuseppe Melacini *[a,b]

Supplementary Figures

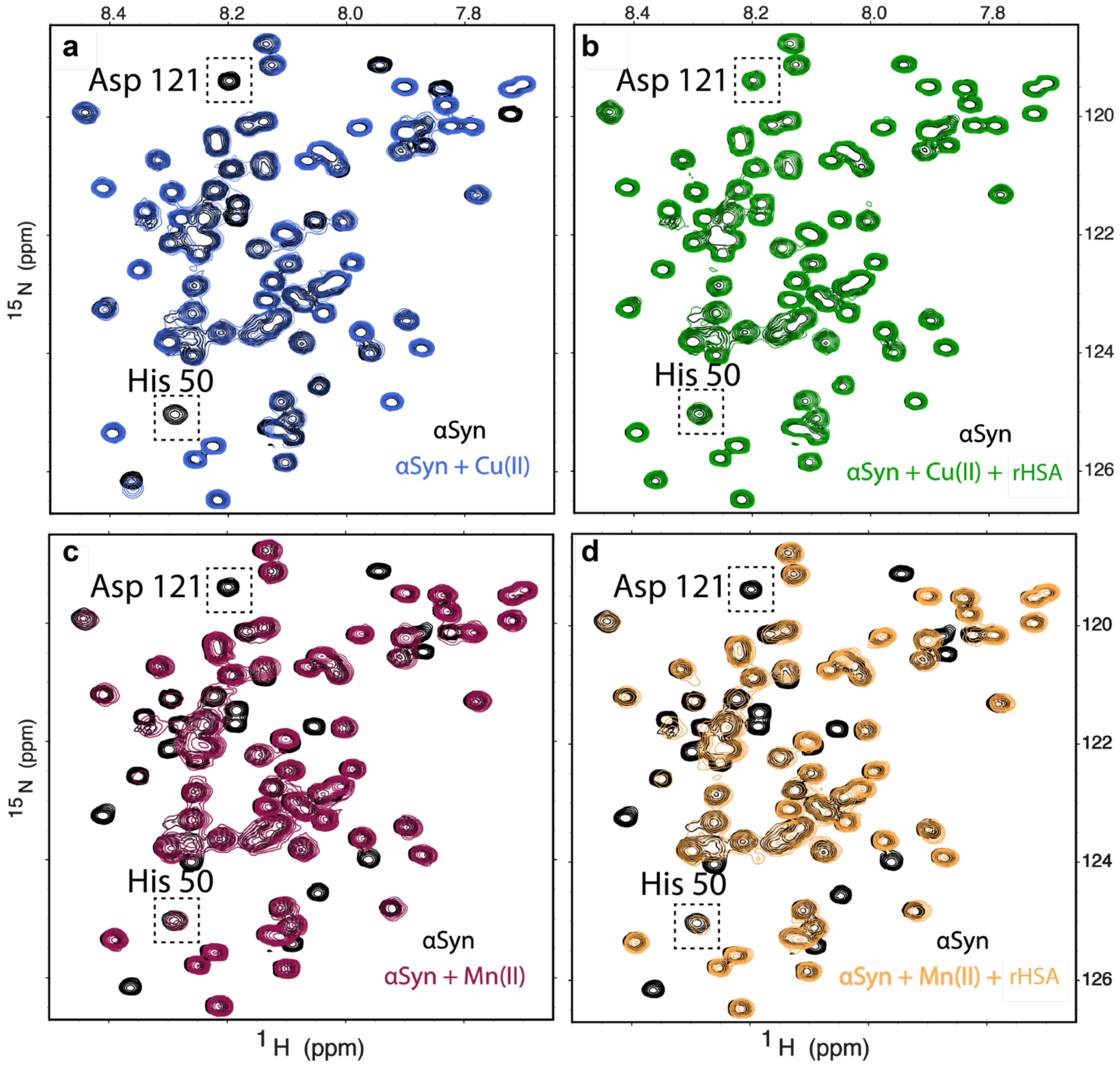

Figure S1. HSA sequesters Cu(II) ions away from αSyn, but not Mn(II) ions. Overlaid contour plots of the ^{1}H-^{15}N HSQC spectra of 60µM Ac-αSyn (black) in presence of (**a**) 60µM Cu(II) (blue), (**b**) 60µM Cu(II) and 60µM rHSA (green), (**c**) 60µM Mn(II) (maroon), and (**d**) 60µM Mn(II) and 60µM rHSA (orange). Spectra were acquired at 10 °C in 50 mM HEPES, pH 7.4.

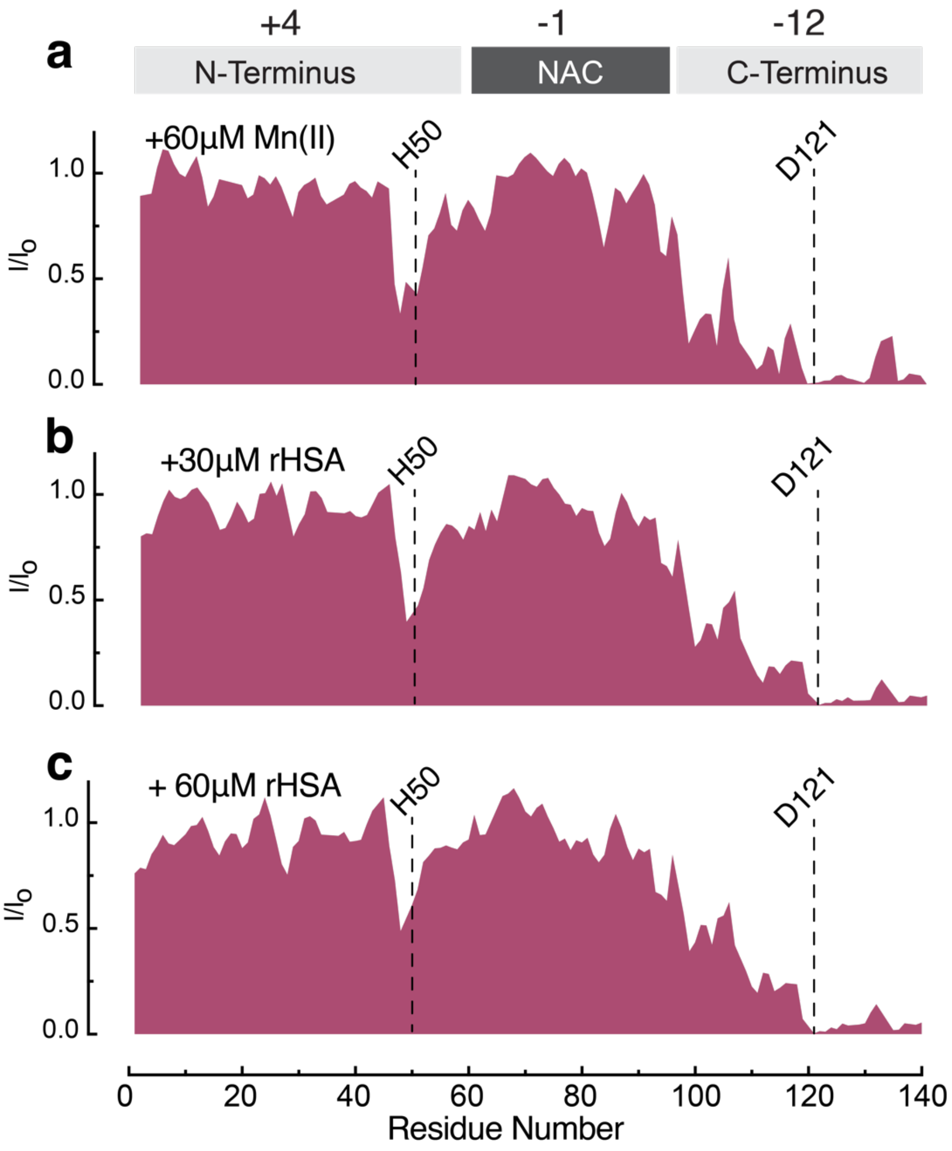

Figure S2. HSA is unable to chelate Mn(II) from αSyn at equimolar concentrations. (a) Normalized ^1H-^{15}N HSQC cross-peak intensities (I/Io) as a function of residue number of 60 µM Ac-αSyn in the presence of 60 µM Mn(II) . (b, c) As a but in the presence of (b) 30µM and (c) 60µM rHSA. Spectra were acquired at 10 °C in 50 mM HEPES, pH 7.4.

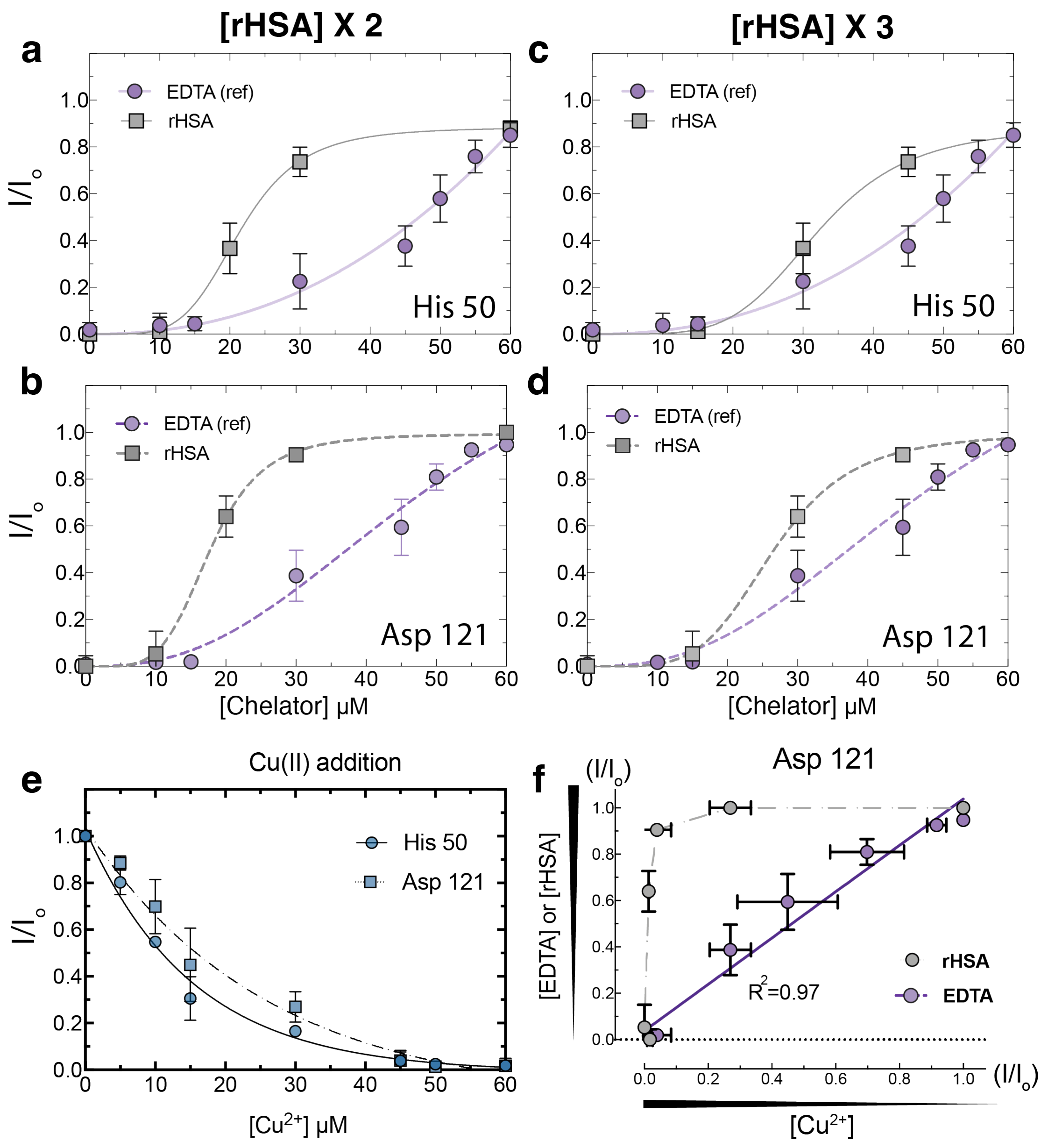

Figure S3. HSA chelates Cu(II) ions more efficiently than the metal chelator EDTA. (a) Isolated I/Io profiles for the binding site His-50 on Ac-αSyn plotted against increased concentrations of rHSA multiplied by a factor of two (grey), and EDTA (same as Figure 2m) as a reference (lilac). (**b**) As (a) but for binding site Asp-121. (**c, d**) As (a, b) but with the rHSA concentration increased by a factor of three. (**e**) Isolated I/Io profiles for binding sites His-50 (solid) and Asp-121 (dashed) of Ac-αSyn upon the addition of increasing concentrations of Cu(II). (**f**) I/Io profiles for αSyn-Cu(II) binding site Asp-121 upon chelation of Cu(II) ions with increasing concentrations of EDTA (lilac) and rHSA (grey) *vs*. the corresponding I/Io ratios observed upon addition to αSyn of an amount of Cu(II) corresponding to the residual αSyn-bound Cu(II), as per panel (e), assuming a binding stoichiometry of 1:1 for Cu(II):EDTA and 2:1 for Cu(II) and rHSA. Spectra were acquired at 10 °C in 50 mM HEPES, pH 7.

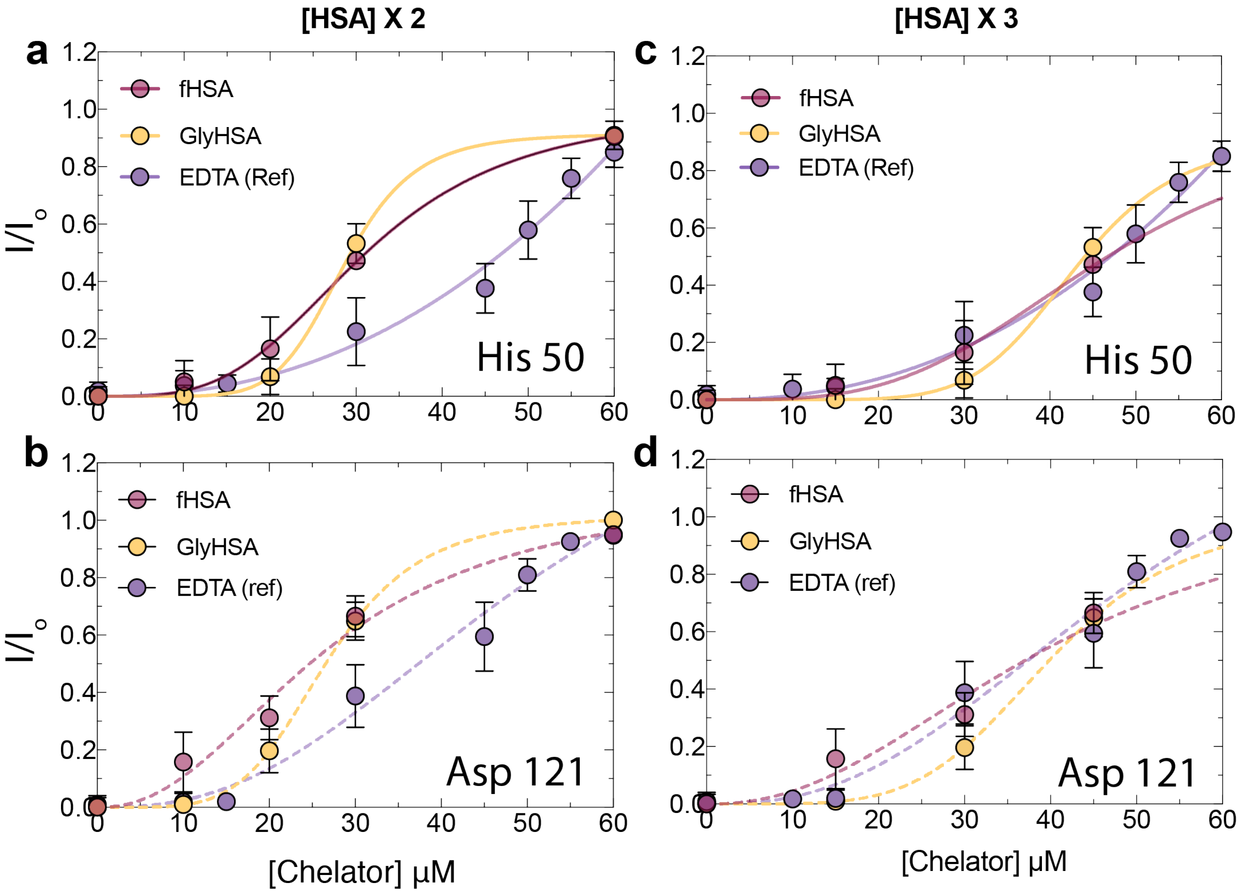

Figure S4 Fatty acid binding and glycation affect the chelating abilities of HSA. (a) Isolated I/Io profiles for the binding site His50 plotted against increasing concentrations of fHSA (red) and Gly rHSA (yellow) multiplied by a factor of two and EDTA (same as Figure 2m) as a reference (lilac). (**b**) As (a) but for binding site Asp-121. (**c, d**) As (a, b) but this time the fHSA and Gly rHSA concentrations were multiplied by a factor of three.

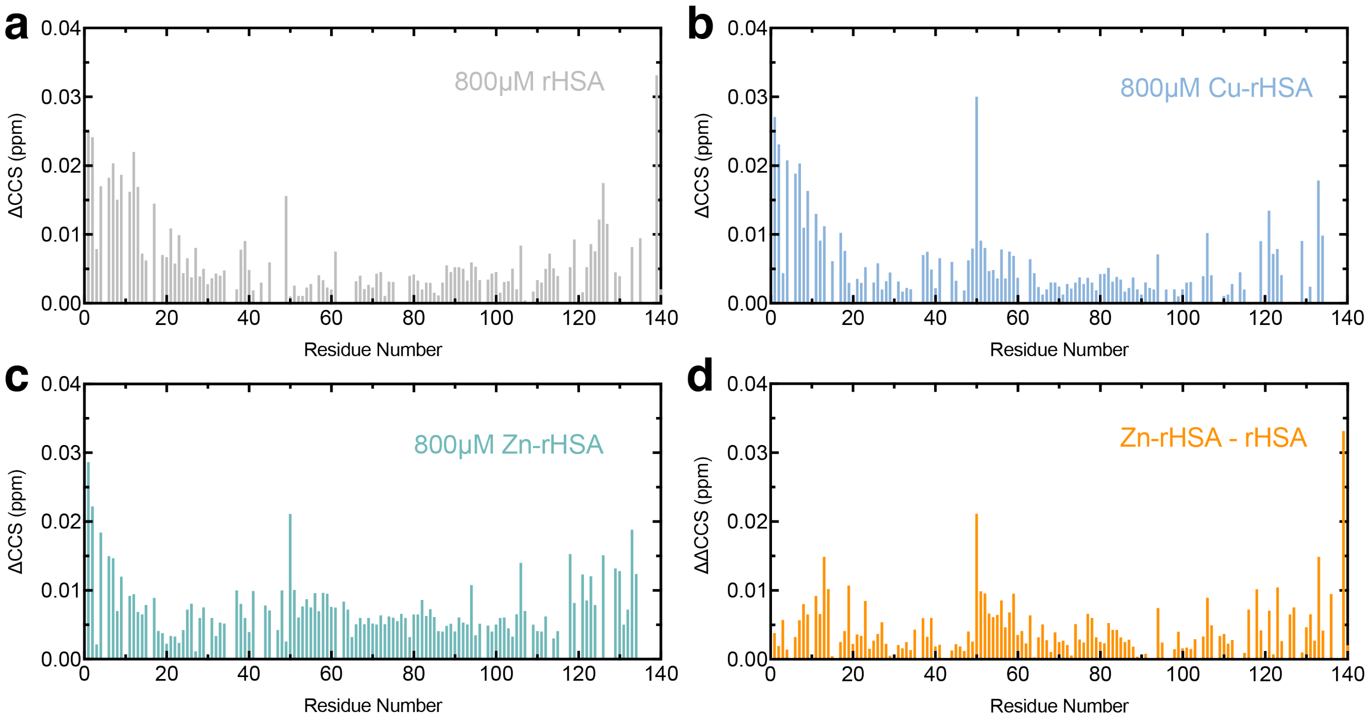

Figure S5. Chemical shift perturbations of 60 μM Ac-αSyn in the presence of (**a**) 800μM rHSA, (**b**) 800 μM Cu(II) and 800 μM rHSA, and (**c**) 800 μM Zn(II) and 800 μM rHSA. (**d**) The difference between the chemical shifts perturbations of 60 μM Ac-αSyn in the presence of 800 μM rHSA *vs.* in the presence of 800 μM Zn(II) and 800 μM rHSA. Spectra were acquired at 10 °C in 50 mM HEPES, pH 7.4.
